# The Competitiveness of Beef Exports From Burkina Faso to Ghana

**DOI:** 10.3389/fvets.2021.619044

**Published:** 2021-08-06

**Authors:** Karl M. Rich, Abdrahmane Wane

**Affiliations:** ^1^International Livestock Research Institute, West Africa Regional Office, Dakar, Senegal; ^2^Department of Agricultural Economics, Ferguson College of Agriculture, Oklahoma State University, Stillwater, OK, United States; ^3^The Mediterranean and Tropical Livestock Systems Joint Research Unit—SELMET (CIRAD, INRAE, SUPAGRO), Université de Montpellier, Montpellier, France

**Keywords:** Ghana, exports, value chain, system dynamics, social accounting matrix, livestock, Burkina Faso

## Abstract

Despite large volumes of cattle stocks in the Sahel, most exports of cattle products remain as live animal sales rather than meat. However, there is increased interest amongst donors and governments to increase value-added exports of beef. In this paper, we provide results from a simulation analysis that explores the prospective competitiveness and benefits of exporting beef from Burkina Faso to Ghana rather than live animals. The paper reviews trading patterns in live animals along the corridor and meat imports from overseas destinations to Ghana. Model results highlight limited competitiveness of the main products demanded in destination markets (offals). Market segmentation strategies, infrastructure development, and animal productivity all generate marginal improvements in competitiveness, but not enough to compete with third-country supplies. Only specific, largely external macroeconomic conditions provide for significant improvements in competitiveness. The paper further reveals the relatively modest employment gains associated with increased exports of meat in lieu of live animals. The analysis suggests a re-think on large-scale investments in downstream functions in the value chain, instead illustrating the fundamental role of upstream investments in productivity, animal health, and collective action to promote greater market integration between pastoralists and formal sector buyers.

## Introduction

In West Africa, there has been a recent renewal of the policy debates associated with the promotion of value-added beef exports in lieu of traditional, largely pastoral-based, trade in live animals from the Sahel to coastal West African countries. These pressures have emerged in part from increased pressure and tensions between pastoral and agricultural communities over land and resources that are exacerbated further by climatic stress. At the same time, the increased dynamism of red meat demand along coastal countries in West Africa has driven a number of planned investments in Sahelian countries (Chad, Mali, Niger, Burkina Faso, Mauritania) to develop export-oriented slaughterhouses that ostensibly will enable these countries to capture more of the value-added associated with the production of livestock.

Demand for red meat is expected to remain strong in West Africa. While this provides an opportunity for African suppliers, it could also pose a major threat if issues of productivity, infrastructure, and quality are not addressed. As noted by Hollinger and Staatz (1), the capacity of ruminant livestock value chains (cattle, sheep, goats) to respond to growing demand for red meat is likely to be constrained by low herd productivity due to poor nutrition resulting from seasonal variation in pasture resources and a weak animal feed industry. Poor productivity is also linked to limited investments by farmers on feed, fodder, and other inputs because of low expected returns from undeveloped markets and poor market integration. More generally, low offtake rates coupled with productivity losses due to animal diseases and seasonal feed shortages compromise the long-term capacity of the livestock sector to meet market expectations. Moreover, with growing demand has come increased competition from exports from non-African sources, often at prices well-below what Sahelian suppliers can provide.

In this paper, we examine these issues and opportunities from a simulation analysis that explores the prospective competitiveness of exporting beef from Burkina Faso to Ghana rather than live animals. We combine the use of system dynamics modeling techniques (2, 3) of upstream and downstream marketing and trade of animals and meat in each country with the use of a social accounting matrix to look more carefully at macroeconomic and especially projected employment effects associated with alternative trading models. The paper begins with a review of trading patterns in live animals along the corridor and meat imports from overseas destinations to Ghana. A discussion of the methodology used to assess the prospects of beef exports from Burkina Faso follows, including model assumptions and data used to calibrate the model. We then report the results of our scenario analysis with the model and provide some insights to better interpret model findings.

## The Landscape of Cattle and Beef Production and Trade in Burkina Faso and Ghana

Burkina Faso is one of the largest producers of live cattle in the Sahel. Official statistics of cattle numbers are somewhat dated, but the most recent figures from DGRESS/MRA (4) revealed cattle stocks of just over 9 million head in 2014, growing from 7.6 million head in 2005. Exports of live animals in the same year were estimated at 344,400 (Table 1). Some 267,000 head of cattle were slaughtered in the formal sector in 2014, yielding 30,137 tons of beef. Given official statistics that estimate offtake rates of about 12%, this suggests over 479,000 animals are either slaughtered for domestic consumption and/or exported informally.

**Table 1:** Exports of live animals from Burkina Faso to regional markets, 2005–2014 (thousand heads).

**Destination country**	**2005**	**2006**	**2007**	**2008**	**2009**	**2010**	**2011**	**2012**	**2013**	**2014**
Benin	10.6	16.9	62.4	122.0	73.2	74.9	70.9	33.7	34.4	35.5
Cote d'Ivoire	44.0	37.6	28.9	27.4	33.6	30.4	34.9	26.2	33.5	56.8
Ghana	90.6	125.7	111.5	93.3	85.2	140.0	152.3	136.0	82.0	82.7
Mali	0.8	2.5	2.0	2.8	1.9	1.0	2.1	2.1	1.3	1.1
Niger	5.6	13.9	12.0	17.7	19.9	14.3	11.7	15.3	15.6	33.8
Nigeria	34.9	60.3	118.6	132.3	101.1	83.4	84.4	138.2	140.4	121.0
Togo	17.1	8.2	21.4	13.3	12.6	12.9	15.5	12.7	9.8	13.5
Others	0.6	0.1	0.2	0.6	0.0	0.1	0.0	0.4	0.5	0.1
Total	204.2	265.2	357.0	409.3	327.6	357.1	371.9	364.6	317.4	344.4

*Direction des Statistiques Sectorielles, Ministère des Ressources Animales (DGESS/MRA) (4), Burkina Faso*.

Movements of cattle between Burkina Faso and Ghana have long been established, with Ghana serving as an important destination market for Burkinabe cattle. Table 1 illustrates the evolution of this trade as reported by official national statistics on animal trade through 2014. Ghana has historically comprised roughly 35–40% of Burkina Faso's exports of cattle, though this share declined propitiously in 2013 and 2014, due in part to the recovery of the market in Cote d'Ivoire after its political crisis in 2011 and an increase in demand from Nigeria.

Despite long-standing trade patterns in animals between Burkina Faso and Ghana, trade in beef has been negligible. Exports of all meats by Burkina Faso (including but not exclusively beef) in the most recent year available from national statistics for disaggregated trade data (2012) reveal exports of just under 143,000 kg, with sales to Ghana only 738 kg (DGESS/MRA).

While imports of beef from Burkina Faso are a negligible portion of consumption in Ghana, imports from other international destinations are particularly important as Ghana is deficit in red meat. It is instructive to first derive consumption volumes in Ghana to contextualize the scale and nature of these imports. MoFA (5) reports animal stocks in Ghana in 2015 of 1.734 million cattle. Simulation results conducted by the authors using DynMod (6) to project herd dynamics in Ghana estimate domestic offtakes of 153,600 animals, which when combined with past imports (82,700 animals) reported in Table 1 suggest total animals available for consumption at 236,300 head of cattle based on older data (2014/2015). According to Suleman (7), around 80–90% of imported animals were from Burkina Faso, while informal reports suggest that total volumes of cattle imports by Ghana are around 100,000 animals per year. These figures would imply that up to a third of animals processed in Ghana for consumption are of Burkinabe origin.^1^ In Ghana and in West Africa in general, it is further important to differentiate between cuts and offals in understanding consumption patterns, as the latter are highly demanded in the region. Using an average carcass weight of 165 kg (based on an average traded animal of 300 kg and carcass yield of 55%), offals comprising 10.4% of the live animal weight, and World Bank estimates of population (29.5 million), national consumption of domestically processed beef cuts is estimated at nearly 39 million kg, while another 7.4 million kg of offals are produced.

International trade data for beef imports by Ghana are inconsistent, with wide variations in the volumes of imports reported by Ghana and exports to Ghana reported by trading partners in the UN Comtrade database. For instance, in 2017, Ghana itself reported imports of frozen beef (HS 0202 of 3.26 million kg, while total exports of global partners to Ghana in that same tariff code were nearly three times this volume (9.13 million kg). Given that the majority of exporters to Ghana in beef are European suppliers with generally reliable statistics, we use this data to estimate Ghana's imports of beef rather than that reported by Ghana to UN Comtrade. This data is summarized in Table 2 during the period 2014–2018 for fresh beef (HS 0201), frozen beef (HS 0202), and offals (HS 0206). While erratic, trends in imports by Ghana are rising, with steady imports of offals (over 30 million kg) during this period and rising imports of frozen beef since 2016.

**Table 2:** Exports of fresh and frozen beef products to Ghana, 2014–2018.

**Year**	**Fresh cuts (HS 0201)**			**Frozen cuts (HS 0202)**			**Offals (HS 0206)**		
	**Value**	**Volume**	**Unit Value**	**Value**	**Volume**	**Unit Value**	**Value**	**Volume**	**Unit Value**
	**(USD)**	**(kg)**	**(USD/kg)**	**(USD)**	**(kg)**	**(USD/kg)**	**(USD)**	**(kg)**	**(USD/kg)**
2014	65,650	5,593	11.74	9,834,838	7,268,323	1.35	31,783,778	30,627,543	1.04
2015	64,185	11,746	5.46	6,794,863	4,202,084	1.62	32,350,555	36,485,517	0.89
2016	202,560	126,388	1.60	12,573,417	9,660,799	1.30	27,335,381	31,375,020	0.87
2017	315,230	246,277	1.28	13,024,312	9,271,383	1.40	31,884,577	33,369,587	0.96
2018	49,388	27,162	1.82	13,279,098	9,976,993	1.33	37,820,374	34,024,121	1.11

*UN Comtrade (updated December 19, 2019)*.

International trade data in meat products do not distinguish between individual cuts in which there can be wide variation in both price and quality. However, implied unit values from individual export suppliers can shed some light on whether imported beef is a primal cut, a low-value cut, a byproduct, or offals. Offals, such as hearts, livers, kidneys, tripe, sinews, etc., are typically priced between US$0.80-US$1.50/kg (f.o.b or c.i.f. depending on product and country of origin). As UN Comtrade data allows the computation of individual supplier unit values for meat exports to Ghana, we can surmise that for beef cuts found in HS 0201 or 0202 where unit values are lower than US$1.50/kg, there is a very high likelihood that such products are some type of by-product and likely sold/consumed alongside offals. In Table 3, we provide disaggregated data from 2018 to derive the share of these products in the import basket of beef imports by Ghana. The data often highlight significant variation in unit value depending on the type of cut (or cuts) sold, though the trade data are not sufficiently granular to tease out specific cuts traded. Those imports that are assumed to be by-products are shaded in gray in Table 3. Our analysis shows that 98% of the volume of beef imports by Ghana was in the form of either low-value byproducts or offals in 2018; while not reported here, a like analysis of 2017 data shows similar results.

**Table 3:** Disaggregation of beef exports to Ghana by country of origin, 2018.

**Country of origin**	**Product**	**Export value (USD)**	**Export volume (kg)**	**Unit value (USD/kg)**
France	Fresh beef	970	128	7.58
Italy	Fresh beef	6,241	227	27.49
Luxembourg	Fresh beef	2,036	54	37.70
South Africa	Fresh beef	4,584	521	8.80
United Kingdom	Fresh beef	32,427	25,867	1.25
USA	Fresh beef	3,057	356	8.59
Botswana	Fresh beef	73	9	8.11
Belgium	Frozen beef	4,202,168	3,291,955	1.28
Brazil	Frozen beef	516,768	316,789	1.63
Canada	Frozen beef	23,390	2,377	9.84
France	Frozen beef	17,203	3,294	5.22
Germany	Frozen beef	182,664	25,222	7.24
Ireland	Frozen beef	3,379,239	2,798,606	1.21
Italy	Frozen beef	655,974	847,603	0.77
Netherlands	Frozen beef	1,264,554	829,678	1.52
Poland	Frozen beef	523,621	572,447	0.91
India	Frozen beef	641,357	317,000	2.02
South Africa	Frozen beef	316,429	47,322	6.69
Spain	Frozen beef	166,833	33,381	5.00
United Kingdom	Frozen beef	1,067,253	844,544	1.26
USA	Frozen beef	289,259	16,475	17.56
Kenya	Frozen beef	20,627	2,300	8.97
Ukraine	Frozen beef	11,759	28,000	0.42
Argentina	Offals	327,316	318,763	1.03
Austria	Offals	31,495	25,600	1.23
Belgium	Offals	4,730,824	3,806,161	1.24
Brazil	Offals	5,039,315	3,650,443	1.38
Croatia	Offals	77,479	125,000	0.62
Cyprus	Offals	22,699	24,660	0.92
Estonia	Offals	29,142	78,000	0.37
France	Offals	484,377	226,367	2.14
Germany	Offals	2,649,137	3,579,817	0.74
Greece	Offals	13,762	24,807	0.55
Iceland	Offals	75,490	98,780	0.76
Ireland	Offals	8,114,371	6,761,650	1.20
Italy	Offals	2,954,252	3,229,267	0.91
Other Asia, nes	Offals	3,746	5,400	0.69
Netherlands	Offals	6,619,382	5,387,134	1.23
Norway	Offals	228,685	248,530	0.92
Paraguay	Offals	84,837	83,997	1.01
Poland	Offals	742,650	904,017	0.82
Russian Federation	Offals	854,487	1,053,160	0.81
Serbia	Offals	76,538	126,580	0.60
South Africa	Offals	2,233	457	4.89
Spain	Offals	1,614,992	1,499,750	1.08
Sweden	Offals	86,789	101,000	0.86
United Kingdom	Offals	2,795,969	2,463,223	1.14
USA	Offals	144,800	174,058	0.83
Ukraine	Offals	15,607	27,500	0.57
TOTAL IMPORTS		51,148,860	44,028,276	1.16
Total low value cuts		11,136,995	9,238,700	1.21
Total offals		37,820,374	34,024,121	1.11
Percentage of low value cuts and offals in total imports		96%	98%	

*UN Comtrade; shaded figures denote low value cuts. See text for details*.

## Materials and Methods

In this paper, we conducted three types of analyses, using data derived from rapid value chain assessments of the trade and marketing dynamics between Burkina Faso and Ghana (7, 8). First, we looked at price gaps and marketing costs between the two countries to explore baseline competitiveness vs. third countries. Second, and expanding on the first analysis, we constructed a system dynamics model of the trade corridor between Burkina Faso and Ghana to explore the long-term marketing and trade dynamics in live animal and meat markets in each country to assess whether value-added sales of meat from Burkina Faso could be competitive vis-à-vis third markets, and under what conditions/scenarios. Third, to explore the broader macroeconomic and employment effects of these different trading alternatives, we employed the most recent social accounting matrix (2013) of Burkina Faso (9) to run multiplier analyses. The latter two methods are described in detail below in turn.

### System Dynamics Model of the Livestock Trade Corridor Between Burkina Faso and Ghana

System dynamics (SD) models are simulation approaches used in the analysis of complex systems. Originally developed in the context of industrial engineering systems, they have been more widely used in a variety of management, ecological, environmental, and social science applications in the last 20 years. SD models move beyond narratives of value chain processes toward frameworks that can provide ex-ante impacts of different investment scenarios associated with technical, marketing, and institutional changes (2). In particular, there could be important feedback effects between the interactions of market dynamics, land use patterns, climate change, institutions, gender dynamics, and socio-economic factors that could influence the uptake and success of any proposed intervention that traditional economic methods or statistical analysis may not pick up or lack local level data to rigorously analyze. In the context of beef trade, such models have been applied in a number of previous analyses including (10) which assessed the viability of a proposed two-stage export certification process in Ethiopia using quarantine stations and feedlots to ensure disease-free status and higher quality of beef for export to markets in the Middle East; a study on commodity-based trade and export feasibility from communal areas of Namibia (11); and an analysis of reforms to improve competitiveness in the beef sector in Botswana (3).

System dynamics models are a set of non-linear differential equations that utilize a graphical programming structure to represent system behavior. They employ core concepts of stocks, flows, and feedbacks in modeling non-linear systems. Stocks represent an accumulation of tangible or intangible goods at time *t*. Flows represent the rate of change of a stock. The net level of a stock changes through flows, either from an inflow into the stock or an outflow out of it. Flows are mediated by parameters which can be a combination of numbers, equations, or graphical functions that regulate the rate of change of inflows or outflows. Feedback denotes the dynamic behavior of a system induced by combinations and interactions of stocks, flows, and parameters. Feedback loops that are reinforcing magnify change in a system, causing either exponential growth or delay, whereas balancing feedback loops converge onto a steady state. SD models typically combine a set of reinforcing and balancing loops. While qualitative archetypes can deliver some intuition about the behavior of simple interactions between combinations of feedback loops, computer simulation is necessary for more complex models (12).

**Figure 1:**
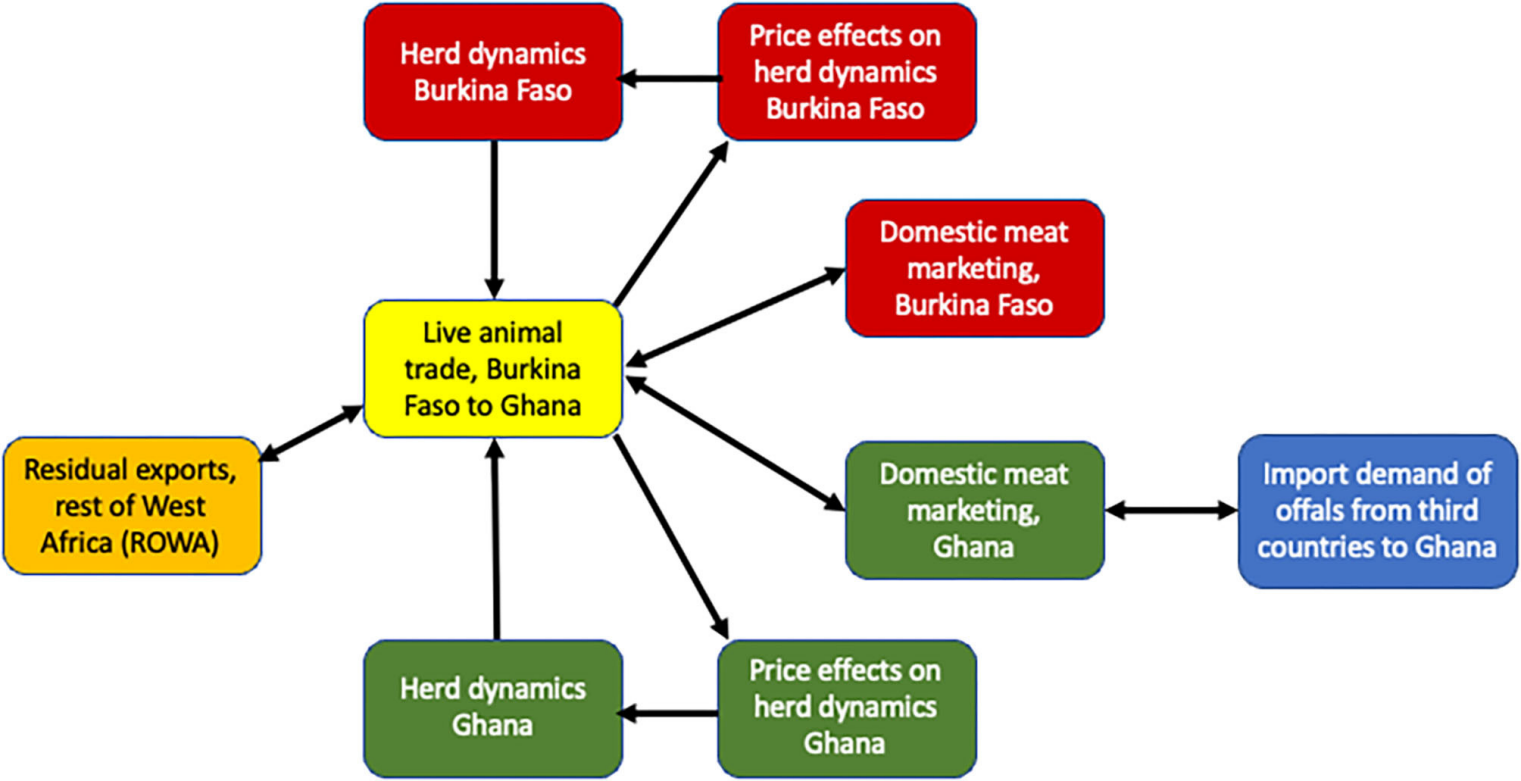
Modules of the simulation model.

The system dynamics model used in this analysis integrates a herd model of animal population dynamics in each country combined with trade dynamics of live animals given excess supply in Burkina Faso and excess demand in Ghana. Downstream, sold animals are then further processed into low-value cuts, high-value cuts, and offals in each market with sales of each depending on consumer demand. Imports of offals into Ghana are also modeled. In Figure 1, the interactions of the different modules of the model are provided. Herd population growth in each country determines the volume of trade in each period, which in turn specifies the price at which trade takes place given excess supply and demand for animals based on the demand for meat in each country. These prices in turn influence the decision of farmers in each country to sell or retain animals in subsequent periods. They also determine how much is traded with other West African countries and, in the case of Ghana, demand for imports from third countries. The specifics of each of these modules is discussed in turn below, with core modules and model equations found in the Supplementary Materials.

**Figure 2:**
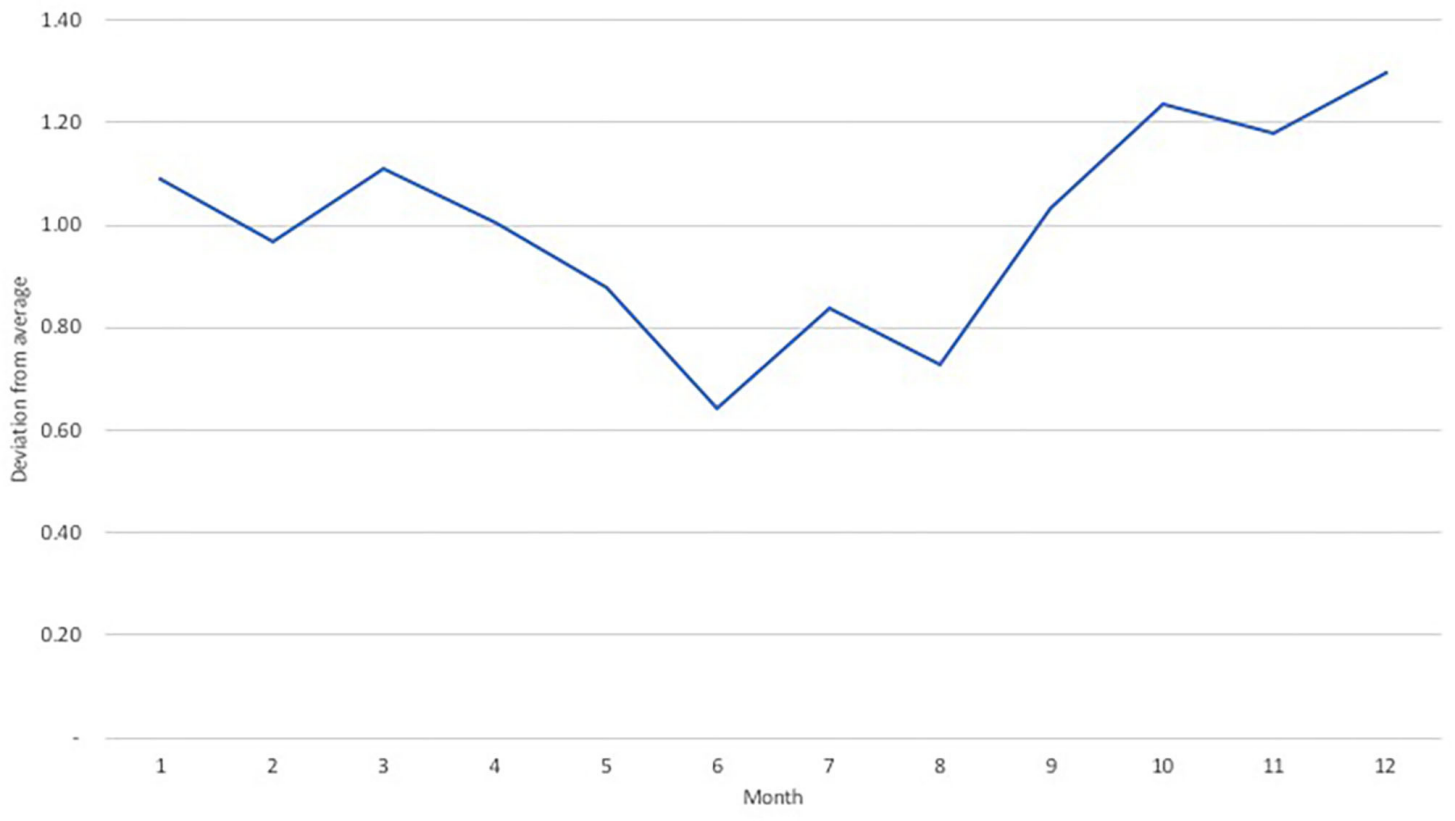
Average seasonality of animal offtakes from Burkina Faso to Ghana, 2014–2018. Ouedraogo (8).

The herd model quantifies the supply of animals available in each market. The herd model is based on DynMod (6), a model developed by CIRAD and ILRI to examine the evolution of herd growth based on parameters of herd demographics, birth and mortality rates, and offtakes for sale. Animals in the herd model are divided into demographic cohorts (juvenile animals, sub-adults, and adults) split by gender; each cohort is represented as a stock in system dynamics. Flows between stocks depend on a set of fixed transition probabilities associated with survival and whether an animal is sold or purchased. The herd model used in this application extends that of Lesnoff (6) in two ways. First, we make offtake rates in both countries price-endogenous to account for supply response based on price changes. We apply a simple double-log functional form with the probability of sales a function of the live animal price. Given that livestock are both consumption (i.e., through their sale) and production (i.e., as inputs for breeding) goods (13), we differentiate our price responsiveness based on age/sex cohorts^2^. For male animals, we assumed elasticities of 0.05 for juvenile animals and 0.1 for sub-adult and adult males, with price elasticities of supply set in proportion to the frequency of sale. For female animals, juveniles are not sold so we set an elasticity of zero for this cohort. Sub-adult females were assumed to have an offtake elasticity of 0.05, while adult females, used for breeding, have an offtake elasticity of −0.05. The latter implies that an increase in price reduces the number of adult females sold so as to breed more animals in future. These low supply elasticity assumptions align with other estimates of live animal figures [see (14)]. Second, we model seasonal offtakes directly based on data reported in Ouedraogo (8). In the original DynMod model, monthly offtake rates are assumed constant and annualized to simulate herd trends on an annual basis. In this version, as the system dynamics model is run on a monthly-time step, we can directly apply a monthly seasonal trend to our price-endogenized offtake equation based on trading patterns from Burkina Faso to Ghana during 2015–2018 (Figure 2).

Net animal offtakes from both countries (representing supply), combined with derived demand for animals based on meat production, define the volumes of animals traded and their price based on an equilibrium relationship between excess supply from Burkina Faso and excess demand from Ghana; in international trade parlance, this is analogous to using a “three-panel” graph [see (15)]. For simplicity, residual live animal sales from Burkina Faso to the rest of West Africa are based on a simple demand function calibrated to derived demand and income growth for Cote d'Ivoire. While Nigeria is an important destination market, a lack of data and fairly stable macroeconomic policy (exchange rate fluctuations) since the large depreciation in 2016 motivated our use of Cote d'Ivoire as a proxy.

The module of live animal trade follows the approach of Sterman (16) which uses inventory relationships to calibrate live animal excess supply and excess demand. System dynamics models of supply and demand derive price and quantity relationships based on the gaps between actual and desired inventory levels, which in turn drive whether prices rise or fall in a particular period. For instance, if actual inventory is greater than desired inventory, this causes pressure to liquidate inventories and reduces the traded price. These prices are transmitted to the model's live animal supply and derived animal demand (marginal cost of meat) functions which then (in the next period) determine a new set of inventory relationships that set subsequent prices (16, 17).

In downstream meat markets, we adopted a long-standing model of joint product pricing under monopoly as first characterized by Colberg (18) in general settings, and Ciriacy-Wantrup (19) in agriculture. This model has been further analyzed by Houck (20), Jensen (21), Manes and Smith (22), and more recently by Shastitko and Shastitko (23), while Piggott and Wohlgenant (24) applied this framework in an international trade setting.

We consider a model of monopoly given that formal sector processing of beef in West Africa tends to be dominated by a very small set of actors (mainly in capital cities) whose actions influence the prices of other informal actors. The motivation for using a monopoly assumption is to address the market power that larger, formal actors have to set prices for animals and meat, which are then transmitted and adopted by smaller, informal actors in both countries. Previous research by Sesay (25) has noted that butcher associations in West Africa typically act as monopolies, and public intervention, particularly downstream in the livestock value chain, has been commonplace. Production and marketing data further bolster this argument. In Burkina Faso, for instance, according to the most recent year (2014) of livestock sector statistics, sales of live animals to the Ouagadougou abattoir averaged 195 head of cattle per day. Assuming 300 days of throughput, this yields 58,500 cattle processed annually, or 6,611 tons of meat [based on a reported 113 kg/animal carcass weight from Ouedraogo (8)]. National statistics further reveal some 102,400 animals were slaughtered in registered slaughter facilities in Ouagadougou in 2014, suggesting that over 57% of animals pass through the main slaughterhouse. In the Accra area of Ghana, Suleman (7) reports that 40% of daily cattle slaughter occurs at the main Accra slaughterhouse. These figures suggest some degree of market power by the main slaughterhouses which justify deviating from a perfect competition assumption. We recognize that while meat processing does not operate as a pure monopoly, neither does it exhibit perfect competition and that a monopoly assumption is a more realistic representation of the actions taken by larger entities with pricing power. An oligopoly representation would be an alternative means of looking at meat markets, though we did not have data to model issues of strategic interaction between firms; this is an area for future research.

**Figure 3:**
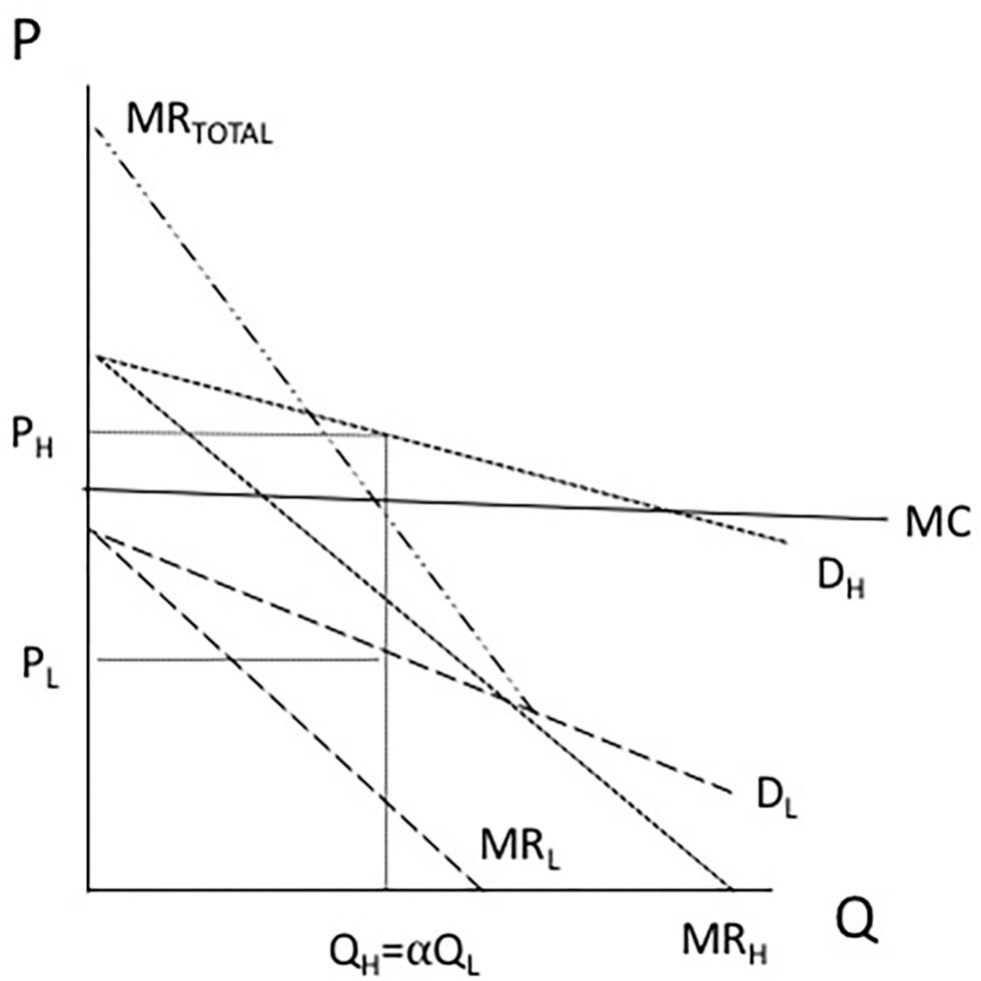
Pricing of joint products under monopoly pricing. Adapted from (18).

The basic model is presented in Figure 3 whereby a monopoly produces two products (here, H denoting high-quality beef and L denoting low-quality beef) in fixed proportions and whereby the marginal costs between them cannot be allocated between their production. In such a model, the monopolist produces where the sum of marginal revenue equals marginal cost, with prices in each market where such quantity intersects the respective demand curve.

**Figure 4:**
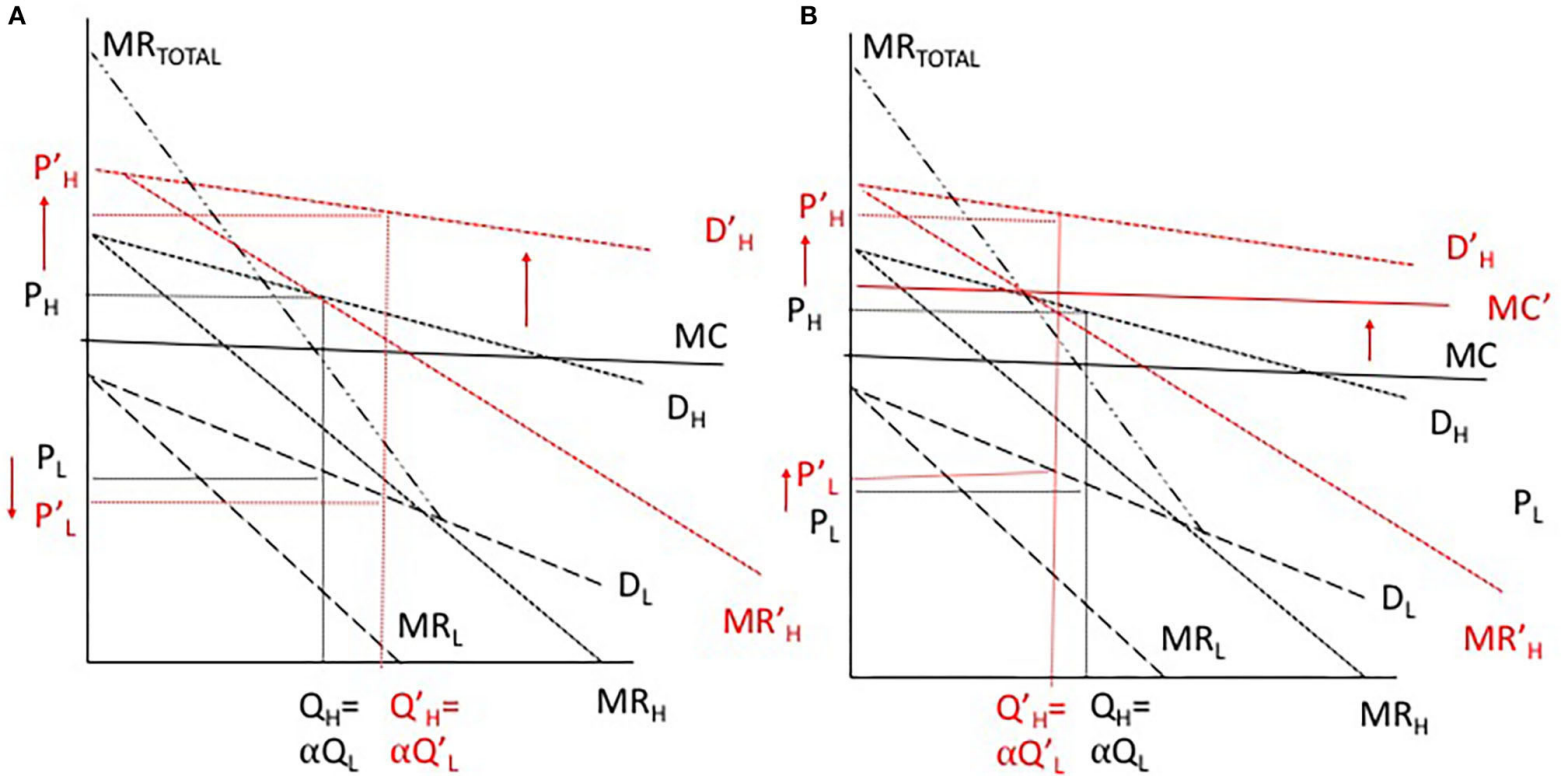
Pricing of joint products under monopoly pricing under scenarios of increased demand **(A)** and increased demand and marginal cost **(B)**. Adapted from (18).

An important consequence of this model, as denoted in Figure 4, is the implication of a change in demand in one of the two products. A shift in the demand curve of H to the right induces a shift of total marginal revenue to the right, causing a rise in the price of H and a fall in the price of L (see the left-panel in Figure 4). Much of the analysis in the articles cited above study the implications whereby such a shift is large enough to cause a glut in the low-value product (L) by virtue of producing where MR_L_ is negative, meaning that a portion of L would be thrown away to maximize monopoly profits.

Our focus with this model is not to consider issues of gluts, but rather to consider the potential tradeoffs that might exist in pursuing higher-value markets. Figure 4A indicates that greater product differentiation would provide more pricing flexibility for L relative to the status quo, but only if the marginal costs of targeting new markets do not change. In Figure 4B, we illustrate that a combination of a shift in demand and a rise in marginal cost to meet such demand may raise the price of the lower-value product, and reduce the pricing flexibility that a monopolist might have. Should scale economies result from greater efficiencies in production that offset the rise in SPS costs, it is possible that marginal costs could fall, providing an opposite effect as that illustrated in Figure 4B.

In the case of Ghana, we also assume the import of offals from third countries. We modeled a simple import demand curve of offals that is a function of the world price of offals, the domestic price of offals, and income. We assume imperfect substitutability of domestic and foreign offals, as the former tend to be fresh and the latter frozen. World prices were assumed to exogenously grow by 2% per year based on the compound annual growth rate of price changes for offals from Table 2 over 2014–2018. We also modeled a modest exchange depreciation of the Cedi against the U.S. dollar and CFA (5% for each), which is lower than the average over 2009–2017 (16% against the USD, 13% against the Euro (CFA).

The model was run monthly over a 10-year period to simulate how herd dynamics influence marketing dynamics, given assumptions on income and demand growth, and to see what options (if any) exist for trade in beef products from Burkina Faso to Ghana. As a system dynamics model, the model does not find an equilibrium in a neoclassical sense, but rather highlights the dynamic evolution of prices, production, and trade on a monthly basis.^3^ As noted below, the model further considers the influence of macroeconomic variables, particularly exchange rate movements, on trade. Supplementary Table 1 summarizes the data and assumptions used in the model and can be found in the Supplementary Materials. The equations for the model can also be found in the Supplementary Materials.

The SD model was used to run the following scenarios. Our first scenario explores the possibility of pursuing higher-value markets to improve pricing flexibility of different cuts to maximize carcass value. The other scenarios chosen highlight some of the key constraints in the beef value chain, that is, low productivity, relatively high marginal costs, and exchange rate volatility as noted in Ouedraogo (8) and Suleman (7).

1. A market segmentation strategy in Burkina Faso, whereby alternative high-value markets are found for higher quality cuts to allow greater pricing flexibility of offals into Ghana;
2. Improving animal productivity by increasing the weight of domestic animals slaughtered in Burkina Faso from 240 to 300 kg;
3. Improving meat processing efficiency through a reduction in the marginal costs of processing in Burkina Faso by 20%;
4. Combinations of the first three scenarios;
5. Scenarios looking at macroeconomic factors, by assuming no depreciation of the Ghanian Cedi against the CFA (but depreciating against the U.S. dollar) – this could be interpreted as both countries adopting the proposed Eco currency.
6. Considering the competitiveness of Burkinabe offals in Northern Ghana, where lower transportation costs would reduce the landed cost of Burkinabe exports and increase the costs of transport of third market offals from the coast to inland markets in Ghana.

### Transport Costs and Margins

To complement the data generated by the SD model, we estimated transport costs between Burkina Faso and Ghana to assess whether prices for offals from Burkina Faso generated by the SD model would be competitive in Ghana with third-country imports after accounting for transportation costs. Teravaninthorn and Raballand (26) estimated transport costs from Ouagadougou to Tema at US$3.53 per km, or US$3,530 given the 1,000 km distance between the two cities. Assuming a 25-ton container of offals at 1,000 CFA^4^/kg (US$1.74/kg at the prevailing exchange rate in 2018), this implies a transportation cost of about 8% of the container value. However, the study by Teravaninthorn and Raballand (26) did not specify whether such costs were for refrigerated transport or not, which would be needed to facilitate such trade. Vilakazi (27) estimated refrigerated transport costs for selected routes in Southern Africa, which ranged from US$0.06/ton/km from Johannesburg to Cape Town to US$0.13/ton/km from Johannesburg to Harare. Braun (28) notes similar costs in South Africa for container transport (US$0.05/ton/km) but small loads have much higher costs (a 5.5-ton van would cost US$0.23/ton/km). Taking the highest of these figures (US$0.13/ton/km) and applying the difference in transport costs between those found by Teravaninthorn and Raballand (26) in West and Southern Africa (52% higher costs in West Africa) yields transport costs of US$4,940 for a 25-ton container of offals, or about 11% of container value. From these ranges, we assume transport costs of 10% for our analysis.

### Social Accounting Matrix Assessment

The other method used in our analysis was the use of a social accounting matrix (or SAM) to quantify prospective macroeconomic and employment effects associated with (a) an expansion of current types of live animal trade and (b) a shift toward meat exports in lieu of live animal exports. A SAM represents a ledger of economic activities within an economy, with such activities specified into accounts that represent aggregates of different sectors, factors of production (labor, capital), and households (29). A SAM is an accounting model whereby the rows of a SAM represent the income received by an account from other accounts, while columns represent expenditures on different accounts; by principles of double-entry accounting, total revenues must equal total expenditures.

SAMs can be transformed into a platform for scenario analysis through the computation of multipliers. A SAM multiplier denotes the economy-wide impact of a one-unit increase in exogenous government spending, investment, or export demand. These multipliers can be aggregated to quantify the total impacts on the value of production output, GDP, or household income. To quantify the impacts of a more specific shock, a matrix of multipliers can be derived. The matrix of multipliers is generated by first computing the SAM's A matrix, where the A matrix comprises the input-output coefficients of the SAM for its endogenous accounts (activities, commodities, factors, and households). Each element of the A matrix, *a*_*ij*_, comes from dividing the corresponding *ij* element of the SAM by the column (*j*) sum. Then, the A matrix is subtracted from an *n X n* identity matrix to generate a matrix (I-A), which is inverted to create a matrix of multipliers, or Leontief inverse (29). Changes to final output can be computed by multiplying the multiplier matrix by an *n X 1* column matrix of final demand (government spending, investment, or export demand) and seeding that matrix with shocks to the appropriate row. To compute changes in export demand for live animals or meat, this entails inputting a value in the relevant commodity row and multiplying that matrix by the multiplier matrix.^5^

In addition, the SAM can be used to compute employment multipliers which show the number of jobs resulting from similar exogenous shocks (30). To do this, we used employment data for Burkina Faso reported by Zidouemba (31) that specified employment by sector aggregate (agriculture, industry, etc.). From the Burkina SAM, we calculated the total wage bill for these aggregated categories and estimated an average aggregate wage by dividing the total wage bill by the number of employees per aggregate category. We then applied the appropriate average wage to the disaggregated SAM accounts to estimate the number of jobs per SAM account. Following ILO (30), we then computed a matrix of employment-output ratios from the SAM accounts (using commodity rows and activity columns of the SAM), which are the number of workers needed to generate 1 million CFA of output. The matrix was multiplied by the relevant partition of the SAM multiplier matrix (commodity rows and activity columns) to generate an employment multiplier matrix, to which our scenarios were applied.

In our SAM analysis, we derived two export demand shocks. We first considered a doubling in the value of live animal exports based on the value found in the 2013 SAM. In the SAM, live cattle exports were estimated at 19.17 billion CFA in 2013, which assuming a value of a live animal of 300,000 CFA suggests live animal exports of nearly 64,000 animals. By contrast, official statistics from Table 1 indicate trade volumes in 2013 were more than five times this figure. To obtain a more realistic indication of an increase in live cattle exports, we took the figure in the SAM and doubled it for exposition. Second, we compute a like shock for meat, where we took an equivalent value of live cattle exports converted to meat based on the yield of products derived from the carcass. We estimated that a 19.17 billion CFA increase in live cattle exports was analogous to 23.09 billion CFA in meat equivalent, based on the value of meat and offals. We used figures from Ouedraogo (8) for live cattle, carcass yield, and offals to estimate these conversion factors.

## Results

### Baseline Competitiveness Assessment

Our results on baseline competitiveness can be found in Tables 4, 5. Our focus is on offals, not cuts, given high demand for such products in Ghana. We estimated the ability of Burkinabe offals to be competitive in Ghana, based on current sales prices, transport prices, and an assessment of competitors. In Table 4, we first estimate the wholesale price of offals from non-Sahel sources based on the FOB prices reported in Table 3 and transport costs, taxes, and margins obtained from Ouedraogo (8) and Suleman (7). Depending on the margin received by the trader, we estimate that average wholesale prices of offals range between US$1.81–1.86/kg (1,043–1,073 CFA/kg); we note this range hides considerable diversity in pricing of different types of offals but gives a plausible indication of the prices for such products.^6^

**Table 4:** Estimates of prices of imported offals in the domestic Ghanaian market.

**Item**	**High trader margin (8%)**	**Low trade margin (5%)**
FOB price imported offals	1.11	1.11
Freight costs (3,500 Euro for 40' container, 25 tons)	0.17	0.17
CIF unit value	1.28	1.28
Tariff (@35%)	0.45	0.45
Trader margin (@min 5%, max 8%)	0.14	0.09
**Wholesale price (USD/kg)**	**1.86**	**1.81**
**Wholesale price (CFA/kg)**	1,073	1,043

*UN Comtrade for FOB prices (refer to Table 3); informant interviews (July 2018) for freight costs and trade margins. Bold value is the sum of the figures above*.

**Table 5:** Comparison of potential Burkinabe offal export prices and Ghanaian domestic prices.

**Item**	**Price**
**Pricing of offals from Burkina Faso**
Price of offals, ex-abattoir Ouagadougou, CFA/kg	1,000
Transport costs (10%), CFA/kg	100
Informal charges (1%), CFA/kg	11
Landed price, CFA/kg	1,111
**Comparative prices in Ghana**
Price paid by butchers in Ghana, CFA/kg equivalent	1,416

*Price and informal charge information compiled from data from Suleman (7) and Ouedraogo (8). Transport costs derived from Vilakazi (24), Braun (25), and Teravaninthorn and Raballand [26]. Prices in Ghana are initially in Ghana Cedis and converted at an exchange rate of 1 Cedi = 117 CFA. See text for details*.

In Table 5, we then posit the export of Burkinabe offals to Ghana, based on current, ex-abattoir prices of offals (1,000 CFA/kg) and an estimate of transportation costs derived from data from Vilakazi (27), Braun (28), and Teravaninthorn and Raballand (26) as noted earlier. Based on these estimates, and informal fees reported in Suleman (7) and Ouedraogo (8), we estimate that the landed wholesale price of fresh Burkinabe offals would be around 1,111 CFA/kg, lower than the price of domestically-produced fresh offals in Ghana (1,416 CFA/kg) but higher than the world prices ranging from 1,043–1,073 CFA/kg reported in Table 4. Even if these Burkinabe prices could be lowered, a number of caveats need to be pointed out, however. First, the acceptability of chilled offals vs. fresh offals in the market is not clear—indications from Suleman (7) and Delavigne (32) are that there is a strong preference for fresh offals and that chilled/frozen products would sell at a discount. Second, the logistical viability of selling chilled offals needs to be explored more thoroughly—Meat and Livestock Australia^7^ note that the shelf life for chilled offals is only about 7 days, and thus exports of chilled offals would require capable logistics that would add costs. Finally, if we consider the potential competitiveness in frozen offals (where such exports are likely more viable), our initial estimates do not consider the added costs of infrastructure (particularly freezing technology) that would be needed for such trade. Given the slight difference in current price gaps, the viability of such trade in frozen offals seems marginal at present, and sensitive to a variety of potential shocks (exchange rates, etc.) that we address in the scenario analysis.

### Scenario Analysis of Alternative Marketing and Trade Protocols

**Figure 5:**
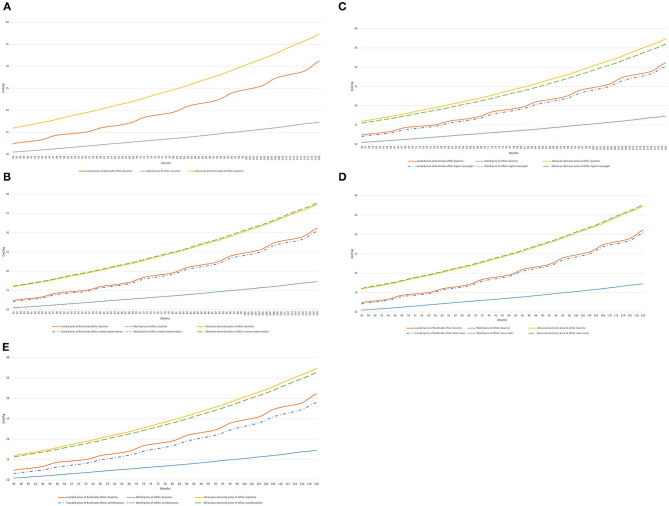
Evolution of offal prices in Burkina Faso, Ghana, and world markets (Cedi/kg) under alternative strategies. **(A)** Baseline. **(B)** A price segmentation strategy. **(C)** Improved animal productivity. **(D)** A reduction in marginal costs in processing in Burkina Faso. **(E)** A combination of strategies **(B)**, **(C)**, and **(D)**. Model simulations.

In Figure 5 through 7, we provide results from our scenario analysis with our SD model. We present results starting from year 3 (month 36) to highlight the steady-state of animal herd dynamics^8^. Figure 5A extrapolates the status-quo scenario given in Tables 4, 5 over the 10-year simulation period, taking into account the adjustment of live animal and meat markets. While Figure 5A shows large gaps in prices between domestically produced offals in Ghana and prospective fresh offals from Burkina Faso, the price of Burkinabe offals is consistently above the price of third-country imports. These gaps widen over time given the relative influences of exchange rate fluctuations, demand growth in both countries, and world price changes.

In Figure 5B through 7, we consider a number of alternative scenarios to explore whether various technical or marketing interventions may improve the status quo situation, with results illustrated against the baseline scenario of Figure 5A.

In the first scenario, we consider the development of high-value markets for high-quality cuts in Burkina Faso. This could be achieved either domestically and/or by sales to third markets. We consider an extreme scenario where Burkina Faso can achieve a price of 4,000 CFA/kg for its high-value cuts (compared to 2,000 CFA/kg in the baseline). Figure 5B reveals that this strategy slightly raises the domestic price in Ghana for offals, as greater demand for meat induced by market segmentation in Burkina Faso reduces the available supply of animals for trade. On the other hand, the price of Burkinabe offals is only slightly lower throughout the simulation period relative to the baseline, thus increasing the price gap between Burkinabe and Ghanaian sourced products. However, as the reduction in the Burkinabe price is modest, it fails to reach more competitive prices with third-country markets.

Improvements in animal productivity result in small reductions in the domestic price of offals in Ghana but have modest effects on the price of Burkinabe offals in the Ghanaian market (Figure 5C). Such a policy has benefits for domestic consumers in both countries for local products, but imported third-country products remain more affordable. Reducing the marginal costs of processing in Burkina Faso (Figure 5D) has slightly counter-intuitive effects. While it lowers the price of Burkinabe offals into Ghana, it very slightly raises the price of domestically produced offals through a similar mechanism as the market segmentation strategy. Namely, reducing marginal costs increases demand for animals in Burkina Faso for processing, lowering availability for trade, and raising the price of live animals. A combined strategy (Figure 5E) reduces prices in both Ghana and Burkina Faso and brings Burkinabe prices closer to third-country prices, but a significant gap still remains.

**Figure 6:**
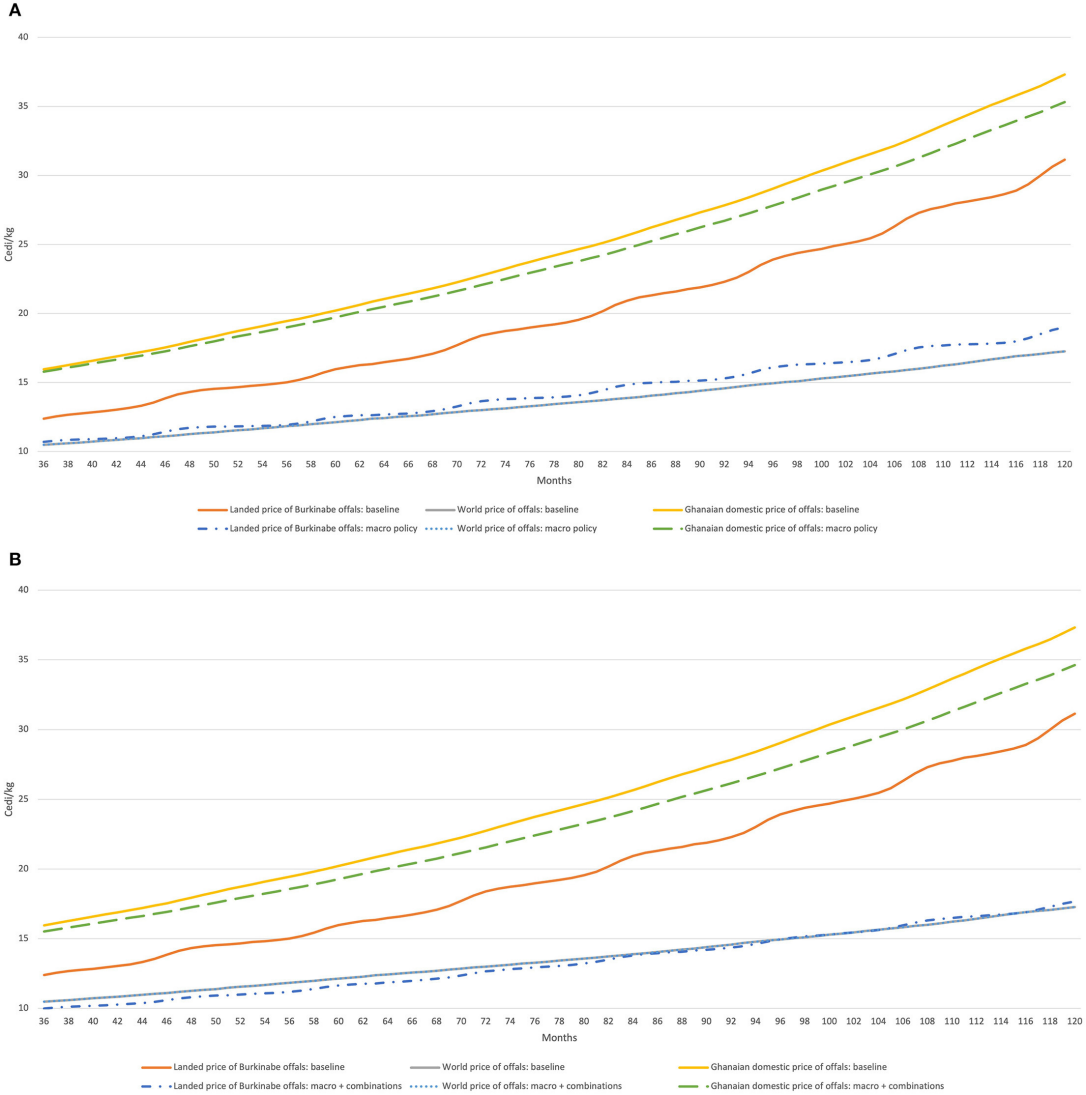
Evolution of offal prices in Burkina Faso, Ghana, and world markets (Cedi/kg) under alternative macroeconomic regimes. **(A)** Baseline plus no depreciation of the Cedi vs. the CFA. **(B)** Macroeconomic changes from **(A)** + combinations of strategies (from Figure 5E). Model simulations.

**Figure 7:**
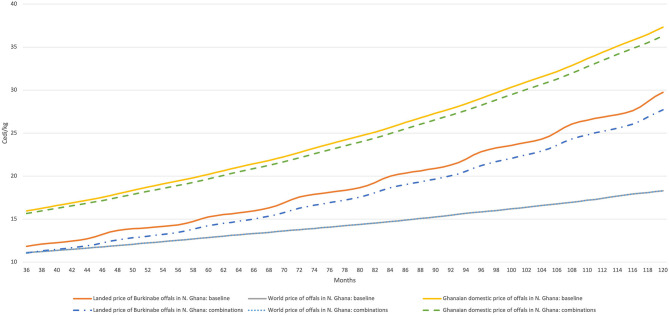
Evolution of offal prices in Burkina Faso, Ghana, and world markets (Cedi/kg) under a combination of strategies—focus on Northern Ghana. Model simulations.

The macroeconomic scenarios in Figure 6 produce perhaps the most interesting results. A stronger Cedi against the CFA brings Burkinabe prices on its own much closer to third-country prices in Cedi terms (Figure 6A). Combining this with the scenarios described above (Figure 6B) enhances Burkina Faso's competitiveness, though policies to make this actionable are largely out of the remit for agricultural ministries. Finally, while Burkinabe offals would be cheaper in Northern Ghana than on the coast, results from Figure 7 highlight a similar, albeit smaller competitiveness gap with third country imports.

### Macroeconomic Impacts of Alternative Trading Scenarios

Results from the SAM analysis can be found in Table 6. We remark that the CFA value of reported findings reflect conditions prevailing in 2013. However, as SAM multipliers are typically robust over several years, using the percentage change of different indicators provides a more interpretable metric that is invariant to the specific base year of the SAM, and will be the focus on the narrative below.

**Table 6:** Scenario analysis of live animal vs. meat exports of output, GDP, employment, and household income.

**Indicator**	**Scenario 1: increased live animal export demand**	**Scenario 2: increased meat export demand**
Change in gross production (million CFA)	43,818 (0.47%)	72,178 (0.77%)
Change in GDP (million CFA)	30,429 (0.57%)	34,845 (0.66%)
Change in employment (# new jobs)	22,938 (0.41%)	26,587 (0.47%)
Change in household income (million CFA)	23,403 (0.49%)	26,940 (0.56%)

*Simulation analysis conducted with the 2013 Burkina Faso SAM (9)*.

*Scenario 1 represents a doubling of the value of live animal exports relative to values reported in the SAM. Scenario 2 converts the shock in scenario 1 to meat equivalent value based on carcass yield and price differentials reported by Ouedraogo (8) and applies this to increased exports of meat (produits d'abattage in the SAM). Gross production, GDP, and household income are reported as percentage changes from gross value (million CFA), while employment is reported as the percentage change in the number of formal jobs. See the text for details*.

The SAM results indicate that higher export demand for meat generates more gross production, GDP, and household income than a similar shock in live animal export demand. However, the difference between the two scenarios is fairly modest. For instance, GDP rises by 0.09% more and household incomes by 0.06% under increased meat demand compared to increased cattle demand. On the other hand, the value of production output rises considerably more (by 0.3%) under a scenario of increase meat export demand, ostensibly given higher multipliers for meat as compared to live cattle. Employment effects are fairly modest in each case. Using 2013 figures, the number of jobs rise by 23,403 under increased live animal demand, and by 26,940 under increased meat demand, a difference of just 3,537 jobs.

## Discussion

From a meso and macroeconomic point of view, competitiveness refers to a broader concept. It is relative to the set of factors enabling a sector to generate growth, contribute to national wealth and improve the standard of living of its inhabitants. This is particularly relevant in the livestock sector in West Africa whose economic contribution, although often underestimated, remains very important. From a value chain perspective, live ruminant exports have shown their resistance to multiple barriers and an adaptability to multifaceted changes. However, very few quantitative studies of regional trade and its relative competitiveness exist for West Africa. This case study on the Burkina Faso-Ghana trade corridor addresses this gap and reveals a number of important findings.

In the context of the long-standing trade between Sahelian and the coastal countries of West Africa, our analysis highlights the persistent lack of competitiveness in prospectively traded beef products (offals) vs. third markets. While there are clear price gaps between the prices of fresh/chilled Burkinabe offals and fresh Ghanaian offals that in the absence of external competition would warrant further promotion, third country imports remain cheaper in coastal markets. None of our proposed scenarios—improved market segmentation, enhanced animal productivity, or reduced processing costs—significantly address those gaps. Those policies are not without merit on their accord. For instance, greater market segmentation will give Burkina Faso more pricing flexibility for its beef in the future, while there are clear upstream benefits to producers and processors in better animal and herd productivity through improved feeding techniques; eradication and control of animal diseases and reduction of pre- and post-production losses. But these policies should be looked at more holistically from the standpoint of improving the livestock and meat sector more generally, and not as a “silver bullet” that yield immediate gains.

If we take the analysis a step further, price differentials may encourage both coastal and Sahelian countries to engage in a non-cooperative game of pursuing infrastructure development. The idea of building slaughterhouses in Sahelian countries is attractive for several reasons. In addition to capturing added value, it makes possible a means to reduce conflicts and potential losses linked to pastoral displacement, improve financial management ratios, create jobs (directly and indirectly in ancillary services), allow countries to converge toward reference health standards, and improve the capacities of actors in the sector. However, this change of paradigm should be carried out in a reasoned manner. Otherwise, their effectiveness and relevance could be severely hampered by insufficient and inadequate supporting infrastructure (poor roads and connectivity; trucks that do not meet standards for the proper conservation and transport of chilled and frozen meat) as well as by governance issues on in the value chain. With such a shift in paradigm, new governance issues emerge including road hassles; changes in sanitary standards for live animals; changes in pricing and marketing mechanisms; the potential transfer of jobs from coastal countries to Sahelian countries; and destruction of other service jobs along the live cattle marketing chain.

Our case study highlights the “curse” of borders in the context of the livestock trade across West Africa. The organization and spatial dimensions of this trade reflect a rational and intrinsic logic based on the resource base and demand amongst participating countries. Similar marketing patterns are found elsewhere amongst major beef suppliers globally. In Argentina, for example, the marketing of cattle has a distinct spatial dimension whereby animals are bred in the drier parts of the north of the country, fattened in the Pampas, and slaughtered in major cities (Buenos Aires, Rosario). The difference in the Argentine case is that the value added of production remains in one country and is not competed with our fought over as it is increasingly in the Sahel and West Africa. The development of innovative institutions fostered by greater regional integration and governance structures that share the benefits of this trade could be one way to better link and foster collaborative actions that sustainably build and grow this value chain.

Although our case study of the Burkina Faso—Ghana trade corridor provides very interesting findings, a broader study of trade dynamics across West Africa would be a useful area for future research. In particular, greater work on the dynamics of markets in and those that serve Nigeria would be critical given its importance as the largest consumption market for the region. Linking such modeling platforms at pan-Sahel level to address substitution effects within and across markets and their dynamics would also be a valuable way forward.

## Conclusions

In this paper, we developed a comprehensive approach to better understand the prospective gains of exporting beef from Burkina Faso to Ghana rather than live animals. Our analysis indicated that while Burkina Faso would be directly competitive in Ghana in meat (offals) given lower prices for offals produced in Burkina Faso, these prices remain higher than third country suppliers, as live animal prices and production costs are generally higher in West Africa. Market segmentation strategies, infrastructure development, and animal productivity all generate marginal improvements in competitiveness, but not enough to displace competitors. Live animal exports remain an important pathway for trade for Sahelian countries like Burkina Faso, and general investments in the sector can both enhance those exports and lead toward a path of greater regional integration to foster value-adding in the sector.

## Data Availability Statement

The original contributions presented in the study are included in the article/Supplementary Material, further inquiries can be directed to the corresponding author/s.

## Author Contributions

KR and AW jointly developed the study design, collected data, and drafted the manuscript. KR constructed the system dynamics model used in the report and conducted the SD and SAM analyses. Both authors contributed to the article and approved the submitted version.

## Conflict of Interest

The authors declare that the research was conducted in the absence of any commercial or financial relationships that could be construed as a potential conflict of interest.

## Publisher's Note

All claims expressed in this article are solely those of the authors and do not necessarily represent those of their affiliated organizations, or those of the publisher, the editors and the reviewers. Any product that may be evaluated in this article, or claim that may be made by its manufacturer, is not guaranteed or endorsed by the publisher.

## Footnotes

^1^It is likely Ghana imports animals from Mali and/or Niger, but data are not available from these markets.

^2^Initial offtake rates based on (6) are 0.1 for juvenile males, 0.2 for subadult males, 0.21 for adult males, zero for juvenile females, and 0.05 for subadult and adult females based on a generic West African herd profile.

^3^In the absence of population or other growth, the model would (and does) converge to a steady state. However, system dynamics models model the price formation process somewhat differently than a neoclassical economic model as noted above.

^4^CFA stands for Communauté Financière Africaine (African Financial Community) and is the predominant currency used in Francophone West Africa. It is pegged to the Euro at 1 Euro = 655.957 CFA.

^5^More details can be found in Sadoulet and de Janvry (29).

^6^VAT of 18% was not applied in our scenarios as it applies equally to Burkinabe and non-Burkinabe sourced products.

^7^https://www.mla.com.au/globalassets/mla-corporate/research-and-development/program-areas/food-safety/pdfs/shelf-life-of-australian-red-meat-2nd-edition.pdfcombinations of strategies.

^8^Typically, from a given set of herd demographic parameters, it takes two to three years in the model for herd growth patterns to reach a steady state, with production figures (and corresponding impacts on prices and trade) reflecting the noise of adjustment in those first few years. In order to focus on the steady state, we present results starting from year three.
