# Supplementary material for: The Competitiveness of Beef Exports From Burkina Faso to Ghana
Source: Front Vet Sci. 2021 Aug 6;8:619044. doi: 10.3389/fvets.2021.619044 (PMC8378806; doi:10.3389/fvets.2021.619044)
Supplement: Supplementary file 1 [file Data_Sheet_1.pdf]

# *Supplementary Material*

## 1 Supplementary Data: System dynamics model

Core modules shown below:

A. Herd dynamics
B. Price dynamics of the herd model
C. Live animal trade between Burkina Faso and Ghana
D. Domestic meat marketing
E. International meat marketing of offals from third countries to Ghana

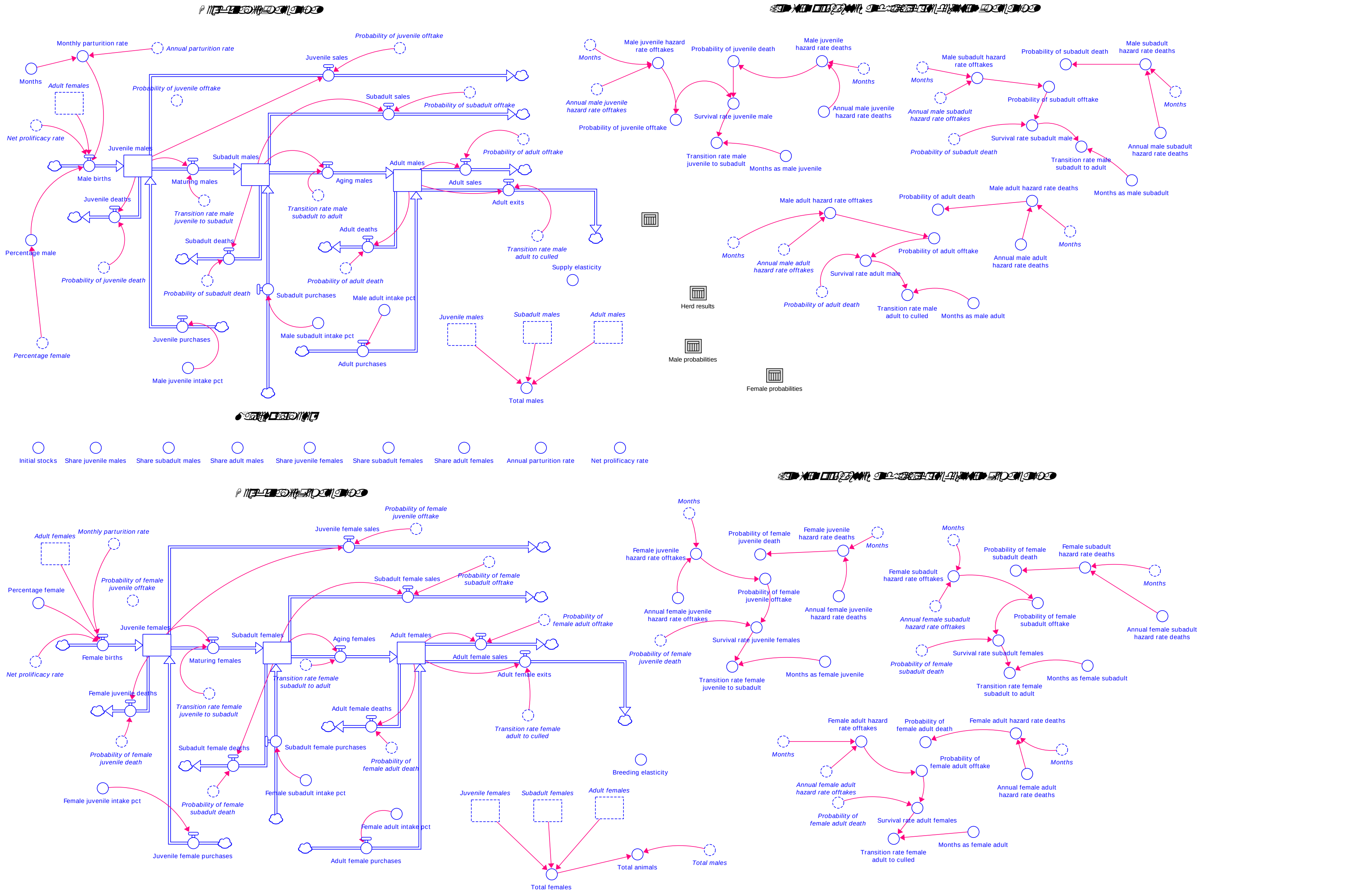


A. Herd demographic model (two models – one per country; the model for Burkina Faso is shown above. The Ghana model is identical)

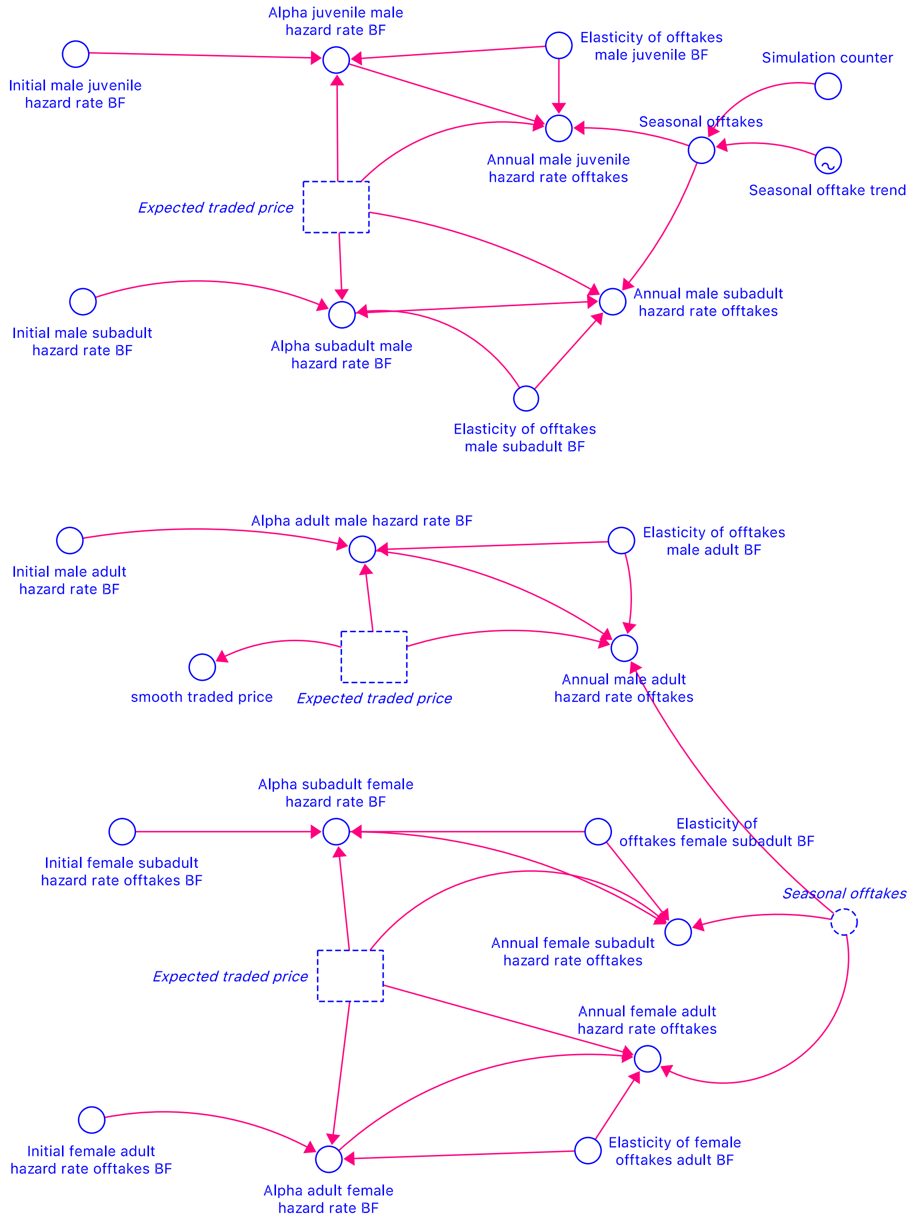


B. Price dynamics of the herd model (shown for Burkina Faso; the Ghana model is identical)

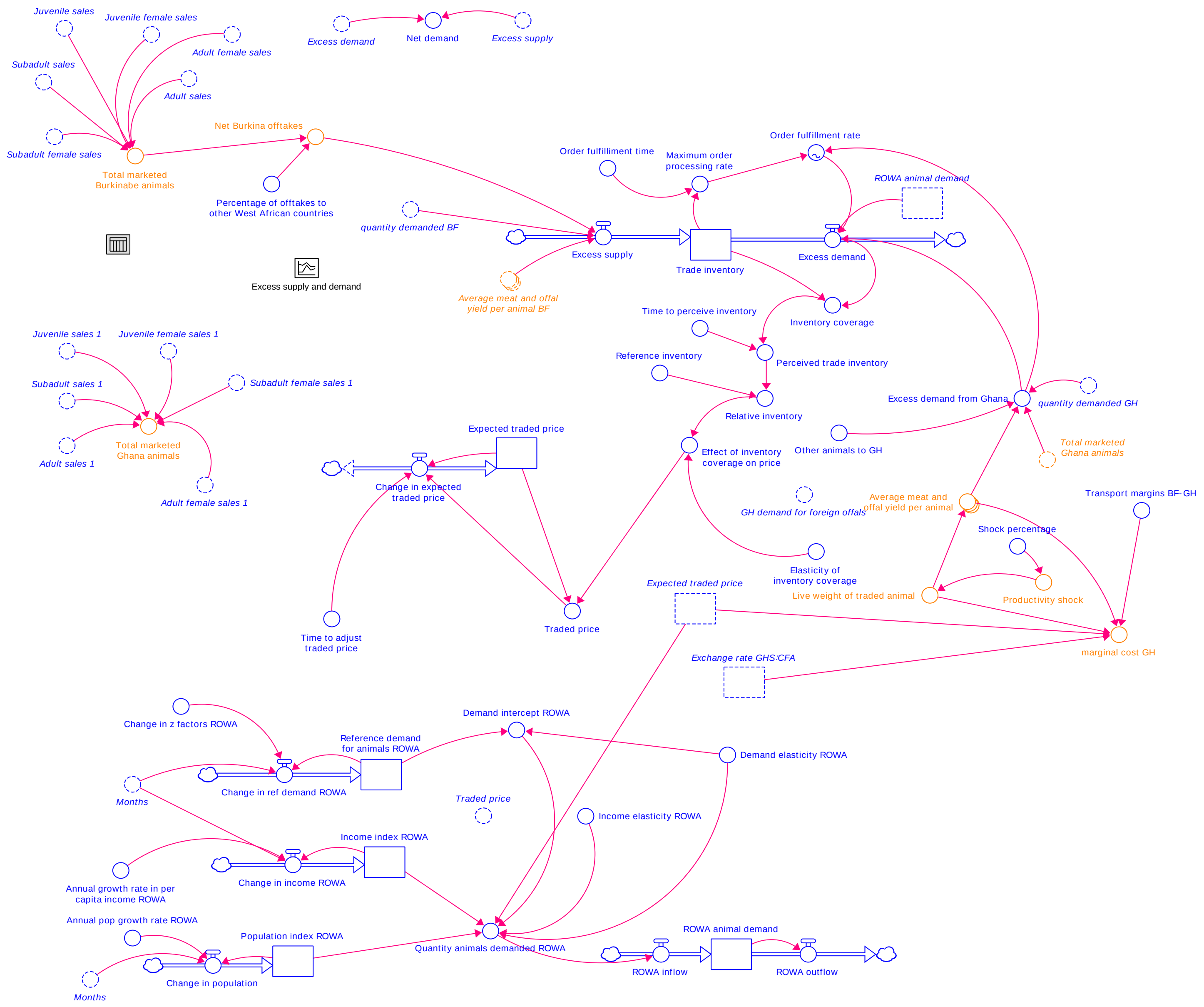


C. Live animal trade between Burkina Faso and Ghana

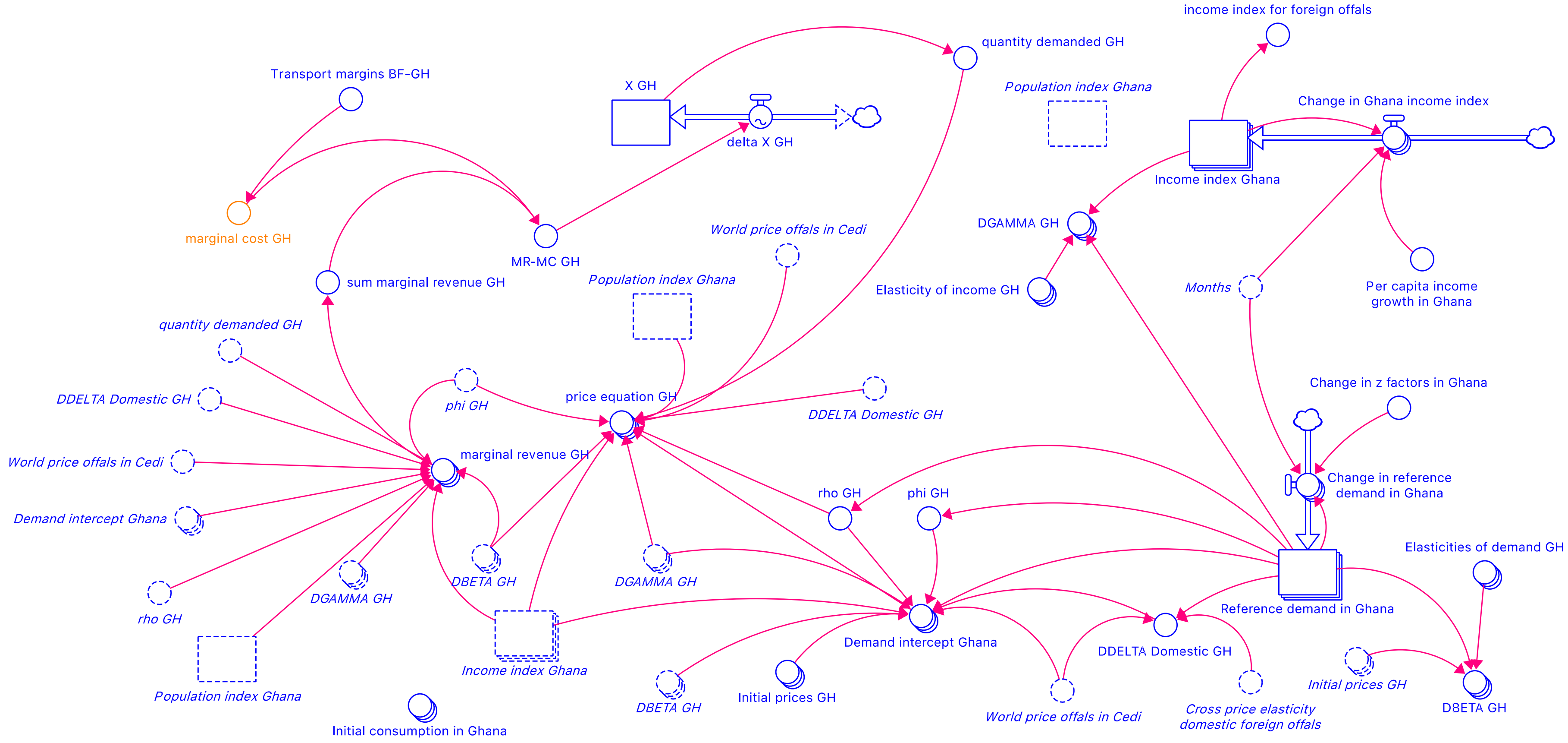


D. Domestic meat marketing model (Ghana model shown; the Burkina Faso model is identical except that there are no imports of offals into Burkina Faso). Parameter naming conventions are based on the Shastitko and Shastitko (2015) paper as noted in the main text.

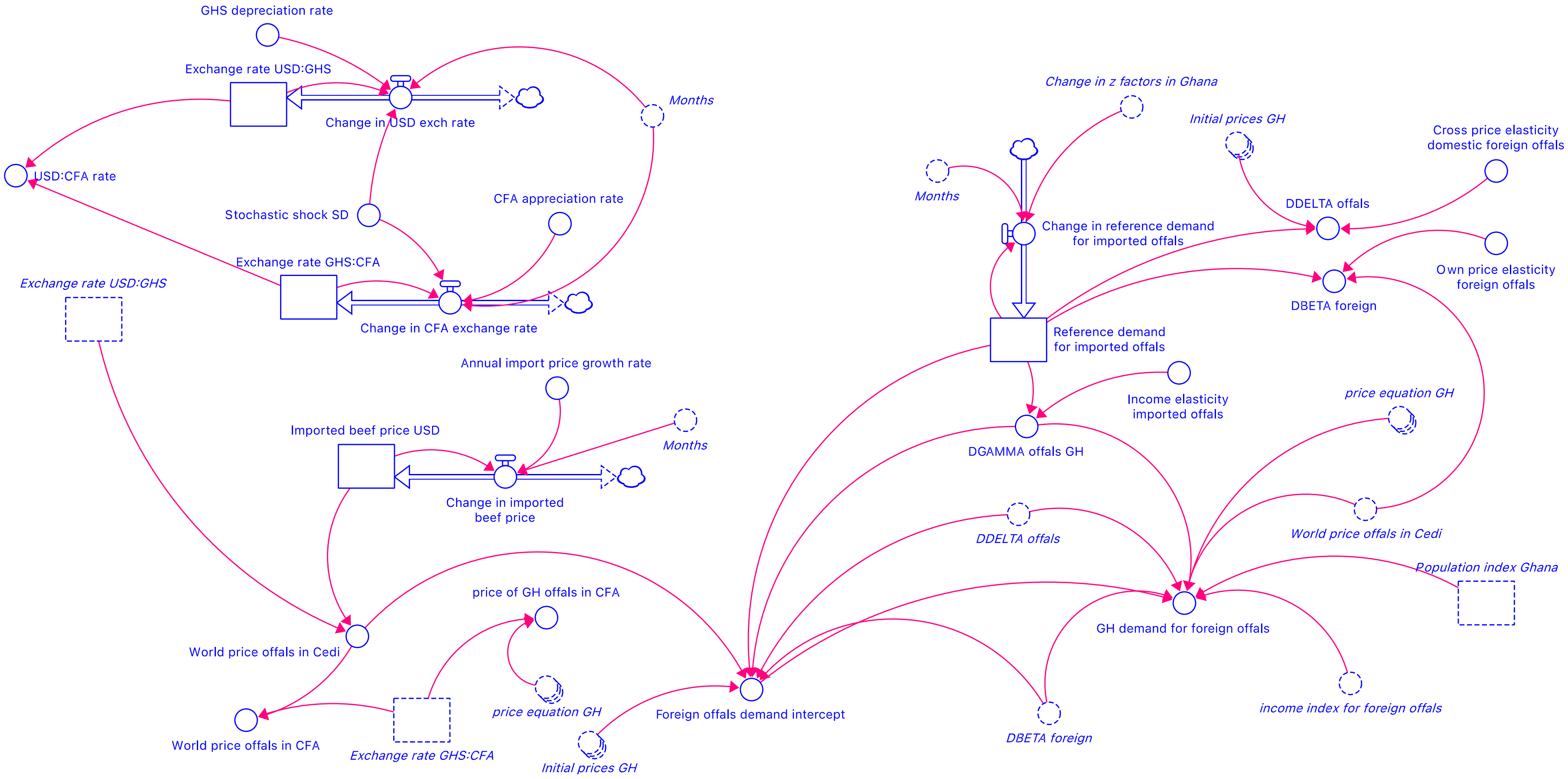


E. International meat marketing of offals from third countries into Ghana

## 2 Supplementary Data: STELLA model code used for analysis

Top-Level Model:

Adult_females(t) = Adult_females(t - dt) + (Aging_females + Adult_female_purchases - Adult_female_deaths - Adult_female_sales - Adult_female_exits) * dt {NON-NEGATIVE}

INIT Adult_females = Share_adult_females*Initial_stocks

INFLOWS:

Aging_females = Subadult_females*Transition_rate_female_subadult_to_adult {UNIFLOW}

Adult_female_purchases = Female_adult_intake_pct {UNIFLOW}

OUTFLOWS:

Adult_female_deaths = Adult_females*Probability_of_female_adult_death {UNIFLOW}

Adult_female_sales = Adult_females*Probability_of_female_adult_offtake {UNIFLOW}

Adult_female_exits = Adult_females*Transition_rate_female_adult_to_culled {UNIFLOW}

Adult_females_1(t) = Adult_females_1(t - dt) + (Aging_females_1 + Adult_female_purchases_1 - Adult_female_deaths_1 - Adult_female_sales_1 - Adult_female_exits_1) * dt {NON-NEGATIVE}

INIT Adult_females_1 = Share_adult_females_1*Initial_stocks_1

INFLOWS:

Aging_females_1 = Subadult_females_1*Transition_rate_female_subadult_to_adult_1 {UNIFLOW}

Adult_female_purchases_1 = Female_adult_intake_pct_1 {UNIFLOW}

OUTFLOWS:

Adult_female_deaths_1 = Adult_females_1*Probability_of_female_adult_death_1 {UNIFLOW}

Adult_female_sales_1 = Adult_females_1*Probability_of_female_adult_offtake_1 {UNIFLOW}

Adult_female_exits_1 = Adult_females_1*Transition_rate_female_adult_to_culled_1 {UNIFLOW}

Adult_males(t) = Adult_males(t - dt) + (Aging_males + Adult_purchases - Adult_deaths - Adult_sales - Adult_exits) * dt {NON-NEGATIVE}

INIT Adult_males = Share_adult_males*Initial_stocks

INFLOWS:

Aging_males = Subadult_males*Transition_rate_male_subadult_to_adult {UNIFLOW}

Adult_purchases = Male_adult_intake_pct {UNIFLOW}

OUTFLOWS:

Adult_deaths = Adult_males*Probability_of_adult_death {UNIFLOW}

Adult_sales = Adult_males*Probability_of_adult_offtake {UNIFLOW}

Adult_exits = Adult_males*Transition_rate_male_adult_to_culled {UNIFLOW}

Adult_males_1(t) = Adult_males_1(t - dt) + (Aging_males_1 + Adult_purchases_1 - Adult_deaths_1 - Adult_sales_1 - Adult_exits_1) * dt {NON-NEGATIVE}

INIT Adult_males_1 = Share_adult_males_1*Initial_stocks_1

INFLOWS:

Aging_males_1 = Subadult_males_1*Transition_rate_male_subadult_to_adult_1 {UNIFLOW}

Adult_purchases_1 = Male_adult_intake_pct_1 {UNIFLOW}

OUTFLOWS:

Adult_deaths_1 = Adult_males_1*Probability_of_adult_death_1 {UNIFLOW}

Adult_sales_1 = Adult_males_1*Probability_of_adult_offtake_1 {UNIFLOW}

Adult_exits_1 = Adult_males_1*Transition_rate_male_adult_to_culled_1 {UNIFLOW}

Annual_female_adult_hazard_rate_offakes_GH(t) = Annual_female_adult_hazard_rate_offakes_GH(t - dt) + (Inflow_FA - Offtake_FA) * dt {NON-NEGATIVE}

INIT Annual_female_adult_hazard_rate_offakes_GH = Initial_female_adult_hazard_rate_offtakes_GH

INFLOWS:

Inflow_FA = Annual_female_adult_hazard_rate_offtakes_2 {UNIFLOW}

OUTFLOWS:

Offtake_FA = DELAY(Annual_female_adult_hazard_rate_offakes_GH, 1) {UNIFLOW}

Annual_female_subadult_hazard_rate_offtakes_GH(t) = Annual_female_subadult_hazard_rate_offtakes_GH(t - dt) + (Inflow_FSA - Outflow_FSA) * dt {NON-NEGATIVE}

INIT Annual_female_subadult_hazard_rate_offtakes_GH = Initial_female_subadult_hazard_rate_offtakes_GH

INFLOWS:

Inflow_FSA = Annual_female_subadult_hazard_rate_offtakes_2 {UNIFLOW}

OUTFLOWS:

Outflow_FSA = DELAY(Annual_female_subadult_hazard_rate_offtakes_GH, 1) {UNIFLOW}

Annual_male_adult_hazard_rate_offtakes_GH(t) = Annual_male_adult_hazard_rate_offtakes_GH(t - dt) + (Inflow_MA_offtake - Outflow_MA_offtake) * dt {NON-NEGATIVE}

INIT Annual_male_adult_hazard_rate_offtakes_GH = Initial_male_adult_hazard_rate_GH

INFLOWS:

Inflow_MA_offtake = Annual_male_adult_hazard_rate_offtakes_2 {UNIFLOW}

OUTFLOWS:

Outflow_MA_offtake = DELAY(Annual_male_adult_hazard_rate_offtakes_GH, 1) {UNIFLOW}

Annual_male_juvenile_hazard_rate_offtakes_GH(t) = Annual_male_juvenile_hazard_rate_offtakes_GH(t - dt) + (Inflow_MJ_offtake - Outflow_MJ_offtake) * dt {NON-NEGATIVE}

INIT Annual_male_juvenile_hazard_rate_offtakes_GH = Initial_male_juvenile_hazard_rate_GH

INFLOWS:

Inflow_MJ_offtake = Annual_male_juvenile_hazard_rate_offtakes_2 {UNIFLOW}

OUTFLOWS:

Outflow_MJ_offtake = DELAY(Annual_male_juvenile_hazard_rate_offtakes_GH, 1) {UNIFLOW}

Annual_male_subadult_hazard_rate_offtakes_GH(t) = Annual_male_subadult_hazard_rate_offtakes_GH(t - dt) + (Inflow_MSA_offtake - Outflow_MSA_offtake) * dt {NON-NEGATIVE}

INIT Annual_male_subadult_hazard_rate_offtakes_GH = Initial_male_subadult_hazard_rate_GH

INFLOWS:

Inflow_MSA_offtake = Annual_male_subadult_hazard_rate_offtakes_2 {UNIFLOW}

OUTFLOWS:

Outflow_MSA_offtake = DELAY(Annual_male_subadult_hazard_rate_offtakes_GH, 1) {UNIFLOW}

Exchange_rate_GHS:CFA(t) = Exchange_rate_GHS:CFA(t - dt) + (Change_in_CFA_exchange_rate) * dt {NON-NEGATIVE}

INIT Exchange_rate_GHS:CFA = 117

INFLOWS:

Change_in_CFA_exchange_rate = Exchange_rate_GHS:CFA*NORMAL(CFA_appreciation_rate/Months, Stochastic_shock_SD, 13)

Exchange_rate_USD:GHS(t) = Exchange_rate_USD:GHS(t - dt) + (Change_in_USD_exch_rate) * dt {NON-NEGATIVE}

INIT Exchange_rate_USD:GHS = 4.6

INFLOWS:

Change_in_USD_exch_rate = Exchange_rate_USD:GHS*NORMAL(GHS_depreciation_rate/Months, Stochastic_shock_SD, 13)

Expected_traded_price(t) = Expected_traded_price(t - dt) + (Change_in_expected_traded_price) * dt {NON-NEGATIVE}

INIT Expected_traded_price = 1000 {CFA per animal}

INFLOWS:

Change_in_expected_traded_price = (Traded_price-Expected_traded_price)/Time_to_adjust_traded_price

Imported_beef_price_USD(t) = Imported_beef_price_USD(t - dt) + (Change_in_imported_beef_price) * dt {NON-NEGATIVE}

INIT Imported_beef_price_USD = 1.86

INFLOWS:

Change_in_imported_beef_price = Imported_beef_price_USD*Annual_import_price_growth_rate/Months

Income_index_BF[Cuts](t) = Income_index_BF[Cuts](t - dt) + (Change_in_BF_income_index[Cuts]) * dt {NON-NEGATIVE}

INIT Income_index_BF[Cuts] = 1

INFLOWS:

Change_in_BF_income_index[Cuts] = Income_index_BF*Per_capita_income_growth_in_BF/Months_3 {UNIFLOW}

Income_index_Ghana[Cuts](t) = Income_index_Ghana[Cuts](t - dt) + (Change_in_Ghana_income_index[Cuts]) * dt {NON-NEGATIVE}

INIT Income_index_Ghana[Cuts] = 1

INFLOWS:

Change_in_Ghana_income_index[Cuts] = Income_index_Ghana*Per_capita_income_growth_in_Ghana/Months {UNIFLOW}

Income_index_ROWA(t) = Income_index_ROWA(t - dt) + (Change_in_income_ROWA) * dt {NON-NEGATIVE}

INIT Income_index_ROWA = 1

INFLOWS:

Change_in_income_ROWA = Income_index_ROWA*Annual_growth_rate_in_per_capita_income_ROWA/Months {UNIFLOW}

Juvenile_females(t) = Juvenile_females(t - dt) + (Female_births + Juvenile_female_purchases - Maturing_females - Female_juvenile_deaths - Juvenile_female_sales) * dt {NON-NEGATIVE}

INIT Juvenile_females = Share_juvenile_females*Initial_stocks-(Adult_females*Monthly_parturition_rate*Net_prolificacy_rate*Percentage_male)

INFLOWS:

Female_births = Adult_females*Net_prolificacy_rate*Monthly_parturition_rate*Percentage_female*(1) {UNIFLOW}

Juvenile_female_purchases = Female_juvenile_intake_pct {UNIFLOW}

OUTFLOWS:

Maturing_females = Juvenile_females*Transition_rate_female_juvenile_to_subadult {UNIFLOW}

Female_juvenile_deaths = Juvenile_females*Probability_of_female_juvenile_death {UNIFLOW}

Juvenile_female_sales = Juvenile_females*Probability_of_female_juvenile_offtake {UNIFLOW}

Juvenile_females_1(t) = Juvenile_females_1(t - dt) + (Female_births_1 + Juvenile_female_purchases_1 - Maturing_females_1 - Female_juvenile_deaths_1 - Juvenile_female_sales_1) * dt {NON-NEGATIVE}

INIT Juvenile_females_1 = Share_juvenile_females_1*Initial_stocks_1-(Adult_females_1*Monthly_parturition_rate_1*Net_prolificacy_rate_1*Percentage_male_1)

INFLOWS:

Female_births_1 = Adult_females_1*Net_prolificacy_rate_1*Monthly_parturition_rate_1*Percentage_female_1*(1) {UNIFLOW}

Juvenile_female_purchases_1 = Female_juvenile_intake_pct_1 {UNIFLOW}

OUTFLOWS:

Maturing_females_1 = Juvenile_females_1*Transition_rate_female_juvenile_to_subadult_1 {UNIFLOW}

Female_juvenile_deaths_1 = Juvenile_females_1*Probability_of_female_juvenile_death_1 {UNIFLOW}

Juvenile_female_sales_1 = Juvenile_females_1*Probability_of_female_juvenile_offtake_1 {UNIFLOW}

Juvenile_males(t) = Juvenile_males(t - dt) + (Male_births + Juvenile_purchases - Maturing_males - Juvenile_deaths - Juvenile_sales) * dt {NON-NEGATIVE}

INIT Juvenile_males = (Share_juvenile_males*Initial_stocks)-(Adult_females*Monthly_parturition_rate*Net_prolificacy_rate*Percentage_male)

INFLOWS:

Male_births = Adult_females*Net_prolificacy_rate*Monthly_parturition_rate*Percentage_male*(1) {UNIFLOW}

Juvenile_purchases = Male_juvenile_intake_pct {UNIFLOW}

OUTFLOWS:

Maturing_males = Juvenile_males*Transition_rate_male_juvenile_to_subadult {UNIFLOW}

Juvenile_deaths = Juvenile_males*Probability_of_juvenile_death {UNIFLOW}

Juvenile_sales = Juvenile_males*Probability_of_juvenile_offtake {UNIFLOW}

Juvenile_males_1(t) = Juvenile_males_1(t - dt) + (Male_births_1 + Juvenile_purchases_1 - Maturing_males_1 - Juvenile_deaths_1 - Juvenile_sales_1) * dt {NON-NEGATIVE}

INIT Juvenile_males_1 = (Share_juvenile_males_1*Initial_stocks_1)-(Adult_females_1*Monthly_parturition_rate_1*Net_prolificacy_rate_1*Percentage_male_1)

INFLOWS:

Male_births_1 = Adult_females_1*Net_prolificacy_rate_1*Monthly_parturition_rate_1*Percentage_male_1*(1) {UNIFLOW}

Juvenile_purchases_1 = Male_juvenile_intake_pct_1 {UNIFLOW}

OUTFLOWS:

Maturing_males_1 = Juvenile_males_1*Transition_rate_male_juvenile_to_subadult_1 {UNIFLOW}

Juvenile_deaths_1 = Juvenile_males_1*Probability_of_juvenile_death_1 {UNIFLOW}

Juvenile_sales_1 = Juvenile_males_1*Probability_of_juvenile_offtake_1 {UNIFLOW}

Population_index_Burkina(t) = Population_index_Burkina(t - dt) + (Change_in_population_BF) * dt {NON-NEGATIVE}

INIT Population_index_Burkina = 1

INFLOWS:

Change_in_population_BF = Population_index_Burkina*Annual_population_growth_rate_BF/Months {UNIFLOW}

Population_index_Ghana(t) = Population_index_Ghana(t - dt) + (Change_in_population_GH) * dt {NON-NEGATIVE}

INIT Population_index_Ghana = 1

INFLOWS:

Change_in_population_GH = Population_index_Ghana*Annual_population_growth_rate_GH/Months {UNIFLOW}

Population_index_ROWA(t) = Population_index_ROWA(t - dt) + (Change_in_population) * dt {NON-NEGATIVE}

INIT Population_index_ROWA = 1

INFLOWS:

Change_in_population = Population_index_ROWA*Annual_pop_growth_rate_ROWA/Months {UNIFLOW}

Reference_demand_for_animals_ROWA(t) = Reference_demand_for_animals_ROWA(t - dt) + (Change_in_ref_demand_ROWA) * dt {NON-NEGATIVE}

INIT Reference_demand_for_animals_ROWA = 340300/12 {represents 37.8% of trade see master database file }

INFLOWS:

Change_in_ref_demand_ROWA = Reference_demand_for_animals_ROWA*Change_in_z_factors_ROWA/Months {UNIFLOW}

Reference_demand_for_imported_offals(t) = Reference_demand_for_imported_offals(t - dt) + (Change_in_reference_demand_for_imported_offals) * dt {NON-NEGATIVE}

INIT Reference_demand_for_imported_offals = Import_demand_for_offals/12

INFLOWS:

Change_in_reference_demand_for_imported_offals = Reference_demand_for_imported_offals*Change_in_z_factors_in_Ghana/Months {UNIFLOW}

Reference_demand_in_BF[Domestic_mixed_cuts](t) = Reference_demand_in_BF[Domestic_mixed_cuts](t - dt) + (Change_in_reference_demand_in_BF[Domestic_mixed_cuts]) * dt {NON-NEGATIVE}

INIT Reference_demand_in_BF[Domestic_mixed_cuts] = Initial_consumption_Burkina[Domestic_mixed_cuts]/12

Reference_demand_in_BF[Domestic_offals](t) = Reference_demand_in_BF[Domestic_offals](t - dt) + (Change_in_reference_demand_in_BF[Domestic_offals]) * dt {NON-NEGATIVE}

INIT Reference_demand_in_BF[Domestic_offals] = Initial_consumption_Burkina[Domestic_offals]/12

Reference_demand_in_BF[Steaks](t) = Reference_demand_in_BF[Steaks](t - dt) + (Change_in_reference_demand_in_BF[Steaks]) * dt {NON-NEGATIVE}

INIT Reference_demand_in_BF[Steaks] = Initial_consumption_Burkina[Steaks]/12

INFLOWS:

Change_in_reference_demand_in_BF[Cuts] = Reference_demand_in_BF*(Change_in_z_factors_in_BF/Months_3) {UNIFLOW}

Reference_demand_in_Ghana[Domestic_mixed_cuts](t) = Reference_demand_in_Ghana[Domestic_mixed_cuts](t - dt) + (Change_in_reference_demand_in_Ghana[Domestic_mixed_cuts]) * dt {NON-NEGATIVE}

INIT Reference_demand_in_Ghana[Domestic_mixed_cuts] = Initial_consumption_in_Ghana[Domestic_mixed_cuts]/12

Reference_demand_in_Ghana[Domestic_offals](t) = Reference_demand_in_Ghana[Domestic_offals](t - dt) + (Change_in_reference_demand_in_Ghana[Domestic_offals]) * dt {NON-NEGATIVE}

INIT Reference_demand_in_Ghana[Domestic_offals] = Initial_consumption_in_Ghana[Domestic_offals]/12

Reference_demand_in_Ghana[Steaks](t) = Reference_demand_in_Ghana[Steaks](t - dt) + (Change_in_reference_demand_in_Ghana[Steaks]) * dt {NON-NEGATIVE}

INIT Reference_demand_in_Ghana[Steaks] = Initial_consumption_in_Ghana[Steaks]/12

INFLOWS:

Change_in_reference_demand_in_Ghana[Cuts] = Reference_demand_in_Ghana*(Change_in_z_factors_in_Ghana/Months) {UNIFLOW}

ROWA_animal_demand(t) = ROWA_animal_demand(t - dt) + (ROWA_inflow - ROWA_outflow) * dt {NON-NEGATIVE}

INIT ROWA_animal_demand = Reference_demand_for_animals_ROWA

INFLOWS:

ROWA_inflow = Quantity_animals_demanded_ROWA {UNIFLOW}

OUTFLOWS:

ROWA_outflow = DELAY(ROWA_animal_demand, DT) {UNIFLOW}

Subadult_females(t) = Subadult_females(t - dt) + (Maturing_females + Subadult_female_purchases - Aging_females - Subadult_female_deaths - Subadult_female_sales) * dt {NON-NEGATIVE}

INIT Subadult_females = Share_subadult_females*Initial_stocks

INFLOWS:

Maturing_females = Juvenile_females*Transition_rate_female_juvenile_to_subadult {UNIFLOW}

Subadult_female_purchases = Female_subadult_intake_pct {UNIFLOW}

OUTFLOWS:

Aging_females = Subadult_females*Transition_rate_female_subadult_to_adult {UNIFLOW}

Subadult_female_deaths = Subadult_females*Probability_of_female_subadult_death {UNIFLOW}

Subadult_female_sales = Subadult_females*Probability_of_female_subadult_offtake {UNIFLOW}

Subadult_females_1(t) = Subadult_females_1(t - dt) + (Maturing_females_1 + Subadult_female_purchases_1 - Aging_females_1 - Subadult_female_deaths_1 - Subadult_female_sales_1) * dt {NON-NEGATIVE}

INIT Subadult_females_1 = Share_subadult_females_1*Initial_stocks_1

INFLOWS:

Maturing_females_1 = Juvenile_females_1*Transition_rate_female_juvenile_to_subadult_1 {UNIFLOW}

Subadult_female_purchases_1 = Female_subadult_intake_pct_1 {UNIFLOW}

OUTFLOWS:

Aging_females_1 = Subadult_females_1*Transition_rate_female_subadult_to_adult_1 {UNIFLOW}

Subadult_female_deaths_1 = Subadult_females_1*Probability_of_female_subadult_death_1 {UNIFLOW}

Subadult_female_sales_1 = Subadult_females_1*Probability_of_female_subadult_offtake_1 {UNIFLOW}

Subadult_males(t) = Subadult_males(t - dt) + (Maturing_males + Subadult_purchases - Aging_males - Subadult_deaths - Subadult_sales) * dt {NON-NEGATIVE}

INIT Subadult_males = Share_subadult_males*Initial_stocks

INFLOWS:

Maturing_males = Juvenile_males*Transition_rate_male_juvenile_to_subadult {UNIFLOW}

Subadult_purchases = Male_subadult_intake_pct {UNIFLOW}

OUTFLOWS:

Aging_males = Subadult_males*Transition_rate_male_subadult_to_adult {UNIFLOW}

Subadult_deaths = Subadult_males*Probability_of_subadult_death {UNIFLOW}

Subadult_sales = Subadult_males*Probability_of_subadult_offtake {UNIFLOW}

Subadult_males_1(t) = Subadult_males_1(t - dt) + (Maturing_males_1 + Subadult_purchases_1 - Aging_males_1 - Subadult_deaths_1 - Subadult_sales_1) * dt {NON-NEGATIVE}

INIT Subadult_males_1 = Share_subadult_males_1*Initial_stocks_1

INFLOWS:

Maturing_males_1 = Juvenile_males_1*Transition_rate_male_juvenile_to_subadult_1 {UNIFLOW}

Subadult_purchases_1 = Male_subadult_intake_pct_1 {UNIFLOW}

OUTFLOWS:

Aging_males_1 = Subadult_males_1*Transition_rate_male_subadult_to_adult_1 {UNIFLOW}

Subadult_deaths_1 = Subadult_males_1*Probability_of_subadult_death_1 {UNIFLOW}

Subadult_sales_1 = Subadult_males_1*Probability_of_subadult_offtake_1 {UNIFLOW}

Trade_inventory(t) = Trade_inventory(t - dt) + (Excess_supply - Excess_demand) * dt {NON-NEGATIVE}

INIT Trade_inventory = Excess_demand_from_Ghana+ROWA_animal_demand

INFLOWS:

Excess_supply = Net_Burkina_offtakes-(quantity_demanded_BF*1000/(Average_meat_and_offal_yield_per_animal_BF[Domestic_mixed_cuts])) {UNIFLOW}

OUTFLOWS:

Excess_demand = (Excess_demand_from_Ghana+ROWA_animal_demand)*Order_fulfillment_rate {UNIFLOW}

X_BF(t) = X_BF(t - dt) + (delta_X_BF) * dt {NON-NEGATIVE}

INIT X_BF = Reference_demand_in_BF[Domestic_mixed_cuts]

INFLOWS:

delta_X_BF = GRAPH("MR-MC_BF")

(-200.0, -1000), (-160.0, -800), (-120.0, -600), (-80.0, -400), (-40.0, -200), (0.0, 0), (40.0, 200), (80.0, 400), (120.0, 600), (160.0, 800), (200.0, 1000)

X_GH(t) = X_GH(t - dt) + (delta_X_GH) * dt {NON-NEGATIVE}

INIT X_GH = Reference_demand_in_Ghana[Domestic_mixed_cuts]

INFLOWS:

delta_X_GH = GRAPH("MR-MC_GH")

(-200.0, -1000), (-160.0, -800), (-120.0, -600), (-80.0, -400), (-40.0, -200), (0.0, 0), (40.0, 200), (80.0, 400), (120.0, 600), (160.0, 800), (200.0, 1000)

Alpha_adult_female_hazard_rate_BF = LN(Initial_female_adult_hazard_rate_offtakes_BF/INIT(Expected_traded_price)^Elasticity_of_female_offtakes_adult_BF)

Alpha_adult_female_hazard_rate_GH = LN(Initial_female_adult_hazard_rate_offtakes_GH/INIT(Expected_traded_price/Exchange_rate_GHS:CFA)^Elasticity_of_female_offtakes_adult_GH)

Alpha_adult_male_hazard_rate_BF = LN(Initial_male_adult_hazard_rate_BF/INIT(Expected_traded_price^Elasticity_of_offtakes_male_adult_BF))

Alpha_adult_male_hazard_rate_GH = LN(Initial_male_adult_hazard_rate_GH/INIT((Expected_traded_price/Exchange_rate_GHS:CFA)^Elasticity_of_offtakes_male_adult_GH))

Alpha_juvenile_male_hazard_rate_BF = LN(Initial_male_juvenile_hazard_rate_BF/INIT(Expected_traded_price^Elasticity_of_offtakes_male_juvenile_BF))

Alpha_juvenile_male_hazard_rate_GH = LN(Initial_male_juvenile_hazard_rate_GH/INIT((Expected_traded_price/Exchange_rate_GHS:CFA)^Elasticity_of_offtakes_male_juvenile_GH))

Alpha_subadult_female_hazard_rate_BF = LN(Initial_female_subadult_hazard_rate_offtakes_BF/INIT(Expected_traded_price)^Elasticity_of_offtakes_female_subadult_BF)

Alpha_subadult_female_hazard_rate_GH = LN(Initial_female_subadult_hazard_rate_offtakes_GH/INIT(Expected_traded_price/Exchange_rate_GHS:CFA)^Elasticity_of_offtakes_female_subadult_GH)

Alpha_subadult_male_hazard_rate_BF =
LN(Initial_male_subadult_hazard_rate_BF/INIT(Expected_traded_price^Elasticity_of_offtakes_male_subadult_BF))

Alpha_subadult_male_hazard_rate_GH =
LN(Initial_male_subadult_hazard_rate_GH/INIT((Expected_traded_price/Exchange_rate_GHS:CFA)^Elasticity_of_offtakes_male_subadult_GH))

Annual_female_adult_hazard_rate_deaths = 0.03

Annual_female_adult_hazard_rate_deaths_1 = 0.03

Annual_female_adult_hazard_rate_offtakes =
EXP(Alpha_adult_female_hazard_rate_BF)*Expected_traded_price^Elasticity_of_female_offtakes_adult_BF*Seasonal_offtakes

Annual_female_adult_hazard_rate_offtakes_2 =
EXP(Alpha_adult_female_hazard_rate_GH)*(Expected_traded_price/Exchange_rate_GHS:CFA)^Elasticity_of_female_offtakes_adult_GH

Annual_female_juvenile_hazard_rate_deaths = 0.13

Annual_female_juvenile_hazard_rate_deaths_1 = 0.13

Annual_female_juvenile_hazard_rate_offtakes = 0

Annual_female_juvenile_hazard_rate_offtakes_1 = 0

Annual_female_subadult_hazard_rate_deaths = 0.05

Annual_female_subadult_hazard_rate_deaths_1 = 0.05

Annual_female_subadult_hazard_rate_offtakes =
EXP(Alpha_subadult_female_hazard_rate_BF)*Expected_traded_price^Elasticity_of_offtakes_female_subadult_BF*Seasonal_offtakes

Annual_female_subadult_hazard_rate_offtakes_2 =
EXP(Alpha_subadult_female_hazard_rate_GH)*(Expected_traded_price/Exchange_rate_GHS:CFA)^Elasticity_of_offtakes_female_subadult_GH

Annual_growth_rate_in_per_capita_income_ROWA = 0.03 {using ten-year average of Cote d'Ivoire from WB}

Annual_import_price_growth_rate = 0.02

Annual_male_adult_hazard_rate_deaths = 0.03

Annual_male_adult_hazard_rate_deaths_1 = 0.03

Annual_male_adult_hazard_rate_offtakes =
EXP(Alpha_adult_male_hazard_rate_BF)*Expected_traded_price^Elasticity_of_offtakes_male_adult_BF*Seasonal_offtakes

Annual_male_adult_hazard_rate_offtakes_2 =
EXP(Alpha_adult_male_hazard_rate_GH)*(Expected_traded_price/Exchange_rate_GHS:CFA)^Elasticity_of_offtakes_male_adult_GH

Annual_male_juvenile_hazard_rate_deaths = 0.13

Annual_male_juvenile_hazard_rate_deaths_1 = 0.13

Annual_male_juvenile_hazard_rate_offtakes =
EXP(Alpha_juvenile_male_hazard_rate_BF)*Expected_traded_price^Elasticity_of_offtakes_male_juvenile_BF*Seasonal_offtakes

Annual_male_juvenile_hazard_rate_offtakes_2 =
EXP(Alpha_juvenile_male_hazard_rate_GH)*(Expected_traded_price/Exchange_rate_GHS:CFA)^Elasticity_of_offtakes_male_juvenile_GH

Annual_male_subadult_hazard_rate_deaths = 0.05

Annual_male_subadult_hazard_rate_deaths_1 = 0.05

Annual_male_subadult_hazard_rate_offtakes =
EXP(Alpha_subadult_male_hazard_rate_BF)*Expected_traded_price^Elasticity_of_offtakes_male_subadult_BF*Seasonal_offtakes

Annual_male_subadult_hazard_rate_offtakes_2 =
EXP(Alpha_subadult_male_hazard_rate_GH)*(Expected_traded_price/Exchange_rate_GHS:CFA)^Elasticity_of_offtakes_male_subadult_GH

Annual_parturition_rate = 0.5

Annual_parturition_rate_1 = 0.5

Annual_pop_growth_rate_ROWA = 0.025

Annual_population_growth_rate_BF = 0.029 {source WB 2017 https://data.worldbank.org/indicator/SP.POP.GROW}

Annual_population_growth_rate_GH = 0.022 {source World Bank 2017 https://data.worldbank.org/indicator/SP.POP.GROW}

Average_meat_and_offal_yield_per_animal[Domestic_mixed_cuts] = 100/240*Live_weight_of_traded_animal

Average_meat_and_offal_yield_per_animal[Domestic_offals] = 25/240*Live_weight_of_traded_animal

Average_meat_and_offal_yield_per_animal[Steaks] = 32/240*Live_weight_of_traded_animal

Average_meat_and_offal_yield_per_animal_BF[Domestic_mixed_cuts] = 100/240*Live_weight_of_sold_animal_BF

Average_meat_and_offal_yield_per_animal_BF[Domestic_offals] = 25/240*Live_weight_of_sold_animal_BF

Average_meat_and_offal_yield_per_animal_BF[Steaks] = 32/240*Live_weight_of_sold_animal_BF

Breeding_elasticity = -0.3

Breeding_elasticity_1 = -0.3

CFA_appreciation_rate = 0

Change_in_z_factors_in_BF = 0

Change_in_z_factors_in_Ghana = 0

Change_in_z_factors_ROWA = 0

Cross_price_elasticity_domestic_foreign_offals = 0.5

DBETA_BF[Domestic_mixed_cuts] = Reference_demand_in_BF[Domestic_mixed_cuts]*Elasticities_of_demand_BF[Domestic_mixed_cuts,Domestic_mixed_cuts]/Initial_prices_BF[Domestic_mixed_cuts]

DBETA_BF[Domestic_offals] = Reference_demand_in_BF[Domestic_offals]*Elasticities_of_demand_BF[Domestic_offals,Domestic_offals]/Initial_prices_BF[Domestic_offals]

DBETA_BF[Steaks] = Reference_demand_in_BF[Steaks]*Elasticities_of_demand_BF[Steaks,Steaks]/Initial_prices_BF[Steaks]

DBETA_foreign = Reference_demand_for_imported_offals*Own_price_elasticity_foreign_offals/INIT(World_price_offals_in_Cedi)

DBETA_GH[Domestic_mixed_cuts] =
Reference_demand_in_Ghana[Domestic_mixed_cuts]*Elasticities_of_demand_GH[Domestic_mixed_cuts,Domestic_mixed_cuts]/Initial_prices_GH[Domestic_mixed_cuts]

DBETA_GH[Domestic_offals] =
Reference_demand_in_Ghana[Domestic_offals]*Elasticities_of_demand_GH[Domestic_offals,Domestic_offals]/Initial_prices_GH[Domestic_offals]

DBETA_GH[Steaks] = Reference_demand_in_Ghana[Steaks]*Elasticities_of_demand_GH[Steaks,Steaks]/Initial_prices_GH[Steaks]

DDELTA_Domestic_GH =
Reference_demand_in_Ghana[Domestic_offals]*Cross_price_elasticity_domestic_foreign_offals/INIT(World_price_offals_in_Cedi)

DDELTA_offals = Reference_demand_for_imported_offals*Cross_price_elasticity_domestic_foreign_offals/Initial_prices_GH[Domestic_offals]

Demand_elasticity_ROWA = -0.5

Demand_intercept_BF[Domestic_mixed_cuts] = (Reference_demand_in_BF[Domestic_mixed_cuts]-DBETA_BF[Domestic_mixed_cuts]*Initial_prices_BF[Domestic_mixed_cuts])-DGAMMA_BF[Domestic_mixed_cuts]*INIT(Income_index_BF[Domestic_mixed_cuts])

Demand_intercept_BF[Domestic_offals] = rho_BF*Reference_demand_in_BF[Domestic_mixed_cuts]-DBETA_BF[Domestic_offals]*Initial_prices_BF[Domestic_offals]-DGAMMA_BF[Domestic_offals]*INIT(Income_index_BF[Domestic_offals])

Demand_intercept_BF[Steaks] = phi_BF*Reference_demand_in_BF[Domestic_mixed_cuts]-DBETA_BF[Steaks]*Initial_prices_BF[Steaks]-DGAMMA_BF[Steaks]*INIT(Income_index_BF[Steaks])

Demand_intercept_Ghana[Domestic_mixed_cuts] = (Reference_demand_in_Ghana[Domestic_mixed_cuts]-DBETA_GH[Domestic_mixed_cuts]*Initial_prices_GH[Domestic_mixed_cuts])-DGAMMA_GH[Domestic_mixed_cuts]*INIT(Income_index_Ghana[Domestic_mixed_cuts])

Demand_intercept_Ghana[Domestic_offals] = rho_GH*Reference_demand_in_Ghana[Domestic_mixed_cuts]-DBETA_GH[Domestic_offals]*Initial_prices_GH[Domestic_offals]-DGAMMA_GH[Domestic_offals]*INIT(Income_index_Ghana[Domestic_offals])-DDELTA_Domestic_GH*INIT(World_price_offals_in_Cedi)

Demand_intercept_Ghana[Steaks] = phi_GH*Reference_demand_in_Ghana[Domestic_mixed_cuts]-DBETA_GH[Steaks]*Initial_prices_GH[Steaks]-DGAMMA_GH[Steaks]*INIT(Income_index_Ghana[Steaks])

Demand_intercept_ROWA = LN(INIT(Reference_demand_for_animals_ROWA)/1000^Demand_elasticity_ROWA)

DGAMMA_BF[Cuts] = Elasticity_of_income_BF[Cuts]*Reference_demand_in_BF[Cuts]/INIT(Income_index_BF[Cuts])

DGAMMA_GH[Cuts] = Elasticity_of_income_GH[Cuts]*Reference_demand_in_Ghana[Cuts]/INIT(Income_index_Ghana[Cuts])

DGAMMA_offals_GH = Income_elasticity_imported_offals*Reference_demand_for_imported_offals

Effect_of_inventory_coverage_on_price = Relative_inventory^Elasticity_of_inventory_coverage

Elasticities_of_demand_BF[Domestic_mixed_cuts, Domestic_mixed_cuts] = -2

Elasticities_of_demand_BF[Domestic_mixed_cuts, Domestic_offals] = 0

Elasticities_of_demand_BF[Domestic_mixed_cuts, Steaks] = 0

Elasticities_of_demand_BF[Domestic_offals, Domestic_mixed_cuts] = 0

Elasticities_of_demand_BF[Domestic_offals, Domestic_offals] = -2

Elasticities_of_demand_BF[Domestic_offals, Steaks] = 0

Elasticities_of_demand_BF[Steaks, Domestic_mixed_cuts] = 0

Elasticities_of_demand_BF[Steaks, Domestic_offals] = 0

Elasticities_of_demand_BF[Steaks, Steaks] = -2

Elasticities_of_demand_GH[Domestic_mixed_cuts, Domestic_mixed_cuts] = -2

Elasticities_of_demand_GH[Domestic_mixed_cuts, Domestic_offals] = 0

Elasticities_of_demand_GH[Domestic_mixed_cuts, Steaks] = 0

Elasticities_of_demand_GH[Domestic_offals, Domestic_mixed_cuts] = 0

Elasticities_of_demand_GH[Domestic_offals, Domestic_offals] = -2

Elasticities_of_demand_GH[Domestic_offals, Steaks] = 0

Elasticities_of_demand_GH[Steaks, Domestic_mixed_cuts] = 0

Elasticities_of_demand_GH[Steaks, Domestic_offals] = 0

Elasticities_of_demand_GH[Steaks, Steaks] = -2

Elasticity_of_female_offtakes_adult_BF = -0.05

Elasticity_of_female_offtakes_adult_GH = -0.05

Elasticity_of_income_BF[Domestic_mixed_cuts] = 1

Elasticity_of_income_BF[Domestic_offals] = 1

Elasticity_of_income_BF[Steaks] = 2

Elasticity_of_income_GH[Domestic_mixed_cuts] = 1

Elasticity_of_income_GH[Domestic_offals] = 1

Elasticity_of_income_GH[Steaks] = 2

Elasticity_of_inventory_coverage = -.5

Elasticity_of_offtakes_female_subadult_BF = 0.05

Elasticity_of_offtakes_female_subadult_GH = 0.05

Elasticity_of_offtakes_male_adult_BF = 0.1

Elasticity_of_offtakes_male_adult_GH = 0.1

Elasticity_of_offtakes_male_juvenile_BF = 0.05

Elasticity_of_offtakes_male_juvenile_GH = 0.05

Elasticity_of_offtakes_male_subadult_BF = 0.1

Elasticity_of_offtakes_male_subadult_GH = 0.1

Excess_demand_from_Ghana = quantity_demanded_GH*1000/(Average_meat_and_offal_yield_per_animal[Domestic_mixed_cuts])-Total_marketed_Ghana_animals-Other_animals_to_GH

Female_adult_hazard_rate_deaths = (IF (Months <= 12) THEN (-LN(1-Annual_female_adult_hazard_rate_deaths)/Months) ELSE (-LN(1-Annual_female_adult_hazard_rate_deaths)/12))

Female_adult_hazard_rate_deaths_1 = (IF (Months_1 <= 12) THEN (-LN(1-Annual_female_adult_hazard_rate_deaths_1)/Months_1) ELSE (-LN(1-Annual_female_adult_hazard_rate_deaths_1)/12))

Female_adult_hazard_rate_offtakes = (IF (Months <= 12) THEN (-LN(1-Annual_female_adult_hazard_rate_offtakes)/Months) ELSE (-LN(1-Annual_female_adult_hazard_rate_offtakes)/12))

Female_adult_hazard_rate_offtakes_1 = (IF (Months_1 <= 12) THEN (-LN(1-Annual_female_adult_hazard_rate_offakes_GH)/Months_1) ELSE (-LN(1-Annual_female_adult_hazard_rate_offakes_GH)/12))

Female_adult_intake_pct = 0

Female_adult_intake_pct_1 = 0

Female_juvenile_hazard_rate_deaths = (IF (Months <= 12) THEN (-LN(1-Annual_female_juvenile_hazard_rate_deaths)/Months) ELSE (-LN(1-Annual_female_juvenile_hazard_rate_deaths)/12))

Female_juvenile_hazard_rate_deaths_1 = (IF (Months_1 <= 12) THEN (-LN(1-Annual_female_juvenile_hazard_rate_deaths_1)/Months_1) ELSE (-LN(1-Annual_female_juvenile_hazard_rate_deaths_1)/12))

Female_juvenile_hazard_rate_offtakes = (IF (Months <= 12) THEN (-LN(1-Annual_female_juvenile_hazard_rate_offtakes)/Months) ELSE (-LN(1-Annual_female_juvenile_hazard_rate_offtakes)/12))

Female_juvenile_hazard_rate_offtakes_1 = (IF (Months_1 <= 12) THEN (-LN(1-Annual_female_juvenile_hazard_rate_offtakes_1)/Months_1) ELSE (-LN(1-Annual_female_juvenile_hazard_rate_offtakes_1)/12))

Female_juvenile_intake_pct = 0

Female_juvenile_intake_pct_1 = 0

Female_subadult_hazard_rate_deaths = (IF (Months <= 12) THEN (-LN(1-Annual_female_subadult_hazard_rate_deaths)/Months) ELSE (-LN(1-Annual_female_subadult_hazard_rate_deaths)/12))

Female_subadult_hazard_rate_deaths_1 = (IF (Months_1 <= 12) THEN (-LN(1-Annual_female_subadult_hazard_rate_deaths_1)/Months_1) ELSE (-LN(1-Annual_female_subadult_hazard_rate_deaths_1)/12))

Female_subadult_hazard_rate_offtakes = (IF (Months <= 12) THEN (-LN(1-Annual_female_subadult_hazard_rate_offtakes)/Months) ELSE (-LN(1-Annual_female_subadult_hazard_rate_offtakes)/12))

Female_subadult_hazard_rate_offtakes_1 = (IF (Months_1 <= 12) THEN (-LN(1-Annual_female_subadult_hazard_rate_offtakes_GH)/Months_1) ELSE (-LN(1-Annual_female_subadult_hazard_rate_offtakes_GH)/12))

Female_subadult_intake_pct = 0

Female_subadult_intake_pct_1 = 0

Foreign_offals_demand_intercept = Reference_demand_for_imported_offals-DBETA_foreign*INIT(World_price_offals_in_Cedi)-DDELTA_offals*Initial_prices_GH[Domestic_offals]-DGAMMA_offals_GH*1

GH_demand_for_foreign_offals = MAX(Population_index_Ghana*income_index_for_foreign_offals*(Foreign_offals_demand_intercept+DBETA_foreign*World_price_offals_in_Cedi+DDELTA_offals*price_equation_GH[Domestic_offals]+DGAMMA_offals_GH*income_index_for_foreign_offals), 0)

GHS_depreciation_rate = 0.05

Import_demand_for_offals = 8946+30054

Income_elasticity_imported_offals = 1

Income_elasticity_ROWA = 1

income_index_for_foreign_offals = Income_index_Ghana[Domestic_offals]

Initial_consumption_Burkina[Domestic_mixed_cuts] = 47855 {51752 old}

Initial_consumption_Burkina[Domestic_offals] = 11964 {12938 old}

Initial_consumption_Burkina[Steaks] = 14835 {16043 old}

Initial_consumption_in_Ghana[Domestic_mixed_cuts] = 29538 {27076 obtained using 275 kg live weight, old figure 23630, 29538 for 300kg}

Initial_consumption_in_Ghana[Domestic_offals] = 7384 {6769 old figure 5908, max 7384}

Initial_consumption_in_Ghana[Steaks] = 9552 {8664 old figure 7562,  9552}

Initial_female_adult_hazard_rate_offtakes_BF = 0.05

Initial_female_adult_hazard_rate_offtakes_GH = 0.05

Initial_female_subadult_hazard_rate_offtakes_BF = 0.05

Initial_female_subadult_hazard_rate_offtakes_GH = 0.05

Initial_male_adult_hazard_rate_BF = .21

Initial_male_adult_hazard_rate_GH = .21

Initial_male_juvenile_hazard_rate_BF = .1

Initial_male_juvenile_hazard_rate_GH = .1

Initial_male_subadult_hazard_rate_BF = .2

Initial_male_subadult_hazard_rate_GH = .2

Initial_prices_BF[Domestic_mixed_cuts] = 2000

Initial_prices_BF[Domestic_offals] = 1000

Initial_prices_BF[Steaks] = 2000

Initial_prices_GH[Domestic_mixed_cuts] = 14.3

Initial_prices_GH[Domestic_offals] = 12.1

Initial_prices_GH[Steaks] = 14.3

Initial_stocks = 10140963  {Extrapolated from official Burkina stats}

Initial_stocks_1 = 1734000

Inventory_coverage = Trade_inventory/Excess_demand

Landed_price_BF_offals_in_Cedi = Landed_price_offals_BF_to_Ghana/Exchange_rate_GHS:CFA

Landed_price_of_offals_to_Northern_Ghana_in_Cedi = price_equation_BF[Domestic_offals]*1.06/Exchange_rate_GHS:CFA

Landed_price_offals_BF_to_Ghana = price_equation_BF[Domestic_offals]*1.11

Live_weight_of_sold_animal_BF = 240

Live_weight_of_traded_animal = 300*Productivity_shock

Male_adult_hazard_rate_deaths = (IF (Months <= 12) THEN (-LN(1-Annual_male_adult_hazard_rate_deaths)/Months) ELSE (-LN(1-Annual_male_adult_hazard_rate_deaths)/12))

Male_adult_hazard_rate_deaths_1 = (IF (Months_1 <= 12) THEN (-LN(1-Annual_male_adult_hazard_rate_deaths_1)/Months_1) ELSE (-LN(1-Annual_male_adult_hazard_rate_deaths_1)/12))

Male_adult_hazard_rate_offtakes = (IF (Months <= 12) THEN (-LN(1-Annual_male_adult_hazard_rate_offtakes)/Months) ELSE (-LN(1-Annual_male_adult_hazard_rate_offtakes)/12))

Male_adult_hazard_rate_offtakes_1 = (IF (Months_1 <= 12) THEN (-LN(1-Annual_male_adult_hazard_rate_offtakes_GH)/Months_1) ELSE (-LN(1-Annual_male_adult_hazard_rate_offtakes_GH)/12))

Male_adult_intake_pct = 0

Male_adult_intake_pct_1 = 0

Male_juvenile_hazard_rate_deaths = (IF (Months <= 12) THEN (-LN(1-Annual_male_juvenile_hazard_rate_deaths)/Months) ELSE (-LN(1-Annual_male_juvenile_hazard_rate_deaths)/12))

Male_juvenile_hazard_rate_deaths_1 = (IF (Months_1 <= 12) THEN (-LN(1-Annual_male_juvenile_hazard_rate_deaths_1)/Months_1) ELSE (-LN(1-Annual_male_juvenile_hazard_rate_deaths_1)/12))

Male_juvenile_hazard_rate_offtakes = (IF (Months <= 12) THEN (-LN(1-Annual_male_juvenile_hazard_rate_offtakes)/Months) ELSE (-LN(1-Annual_male_juvenile_hazard_rate_offtakes)/12))

Male_juvenile_hazard_rate_offtakes_1 = (IF (Months_1 <= 12) THEN (-LN(1-Annual_male_juvenile_hazard_rate_offtakes_GH)/Months_1) ELSE (-LN(1-Annual_male_juvenile_hazard_rate_offtakes_GH)/12))

Male_juvenile_intake_pct = 0

Male_juvenile_intake_pct_1 = 0

Male_subadult_hazard_rate_deaths = (IF (Months <= 12) THEN (-LN(1-Annual_male_subadult_hazard_rate_deaths)/Months) ELSE (-LN(1-Annual_male_subadult_hazard_rate_deaths)/12))

Male_subadult_hazard_rate_deaths_1 = (IF (Months_1 <= 12) THEN (-LN(1-Annual_male_subadult_hazard_rate_deaths_1)/Months_1) ELSE (-LN(1-Annual_male_subadult_hazard_rate_deaths_1)/12))

Male_subadult_hazard_rate_offtakes = (IF (Months <= 12) THEN (-LN(1-Annual_male_subadult_hazard_rate_offtakes)/Months) ELSE (-LN(1-Annual_male_subadult_hazard_rate_offtakes)/12))

Male_subadult_hazard_rate_offtakes_1 = (IF (Months_1 <= 12) THEN (-LN(1-Annual_male_subadult_hazard_rate_offtakes_GH)/Months_1) ELSE (-LN(1-Annual_male_subadult_hazard_rate_offtakes_GH)/12))

Male_subadult_intake_pct = 0

Male_subadult_intake_pct_1 = 0

marginal_cost_BF = 0.4*Expected_traded_price*Live_weight_of_sold_animal_BF/(Average_meat_and_offal_yield_per_animal_BF[Domestic_mixed_cuts]+Average_meat_and_offal_yield_per_animal_BF[Domestic_offals]+Average_meat_and_offal_yield_per_animal_BF[Steaks])*marginal_cost_shocks

marginal_cost_GH = 0.4*Expected_traded_price*"Transport_margins_BF-GH"/Exchange_rate_GHS:CFA*Live_weight_of_traded_animal/(Average_meat_and_offal_yield_per_animal[Domestic_mixed_cuts]+Average_meat_and_offal_yield_per_animal[Domestic_offals]+Average_meat_and_offal_yield_per_animal[Steaks])

marginal_cost_shocks = 1

marginal_revenue_BF[Domestic_mixed_cuts] = Population_index_Burkina*((2*quantity_demanded_BF-Demand_intercept_BF[Domestic_mixed_cuts]-DGAMMA_BF[Domestic_mixed_cuts]*Income_index_BF[Domestic_mixed_cuts])/DBETA_BF[Domestic_mixed_cuts])

marginal_revenue_BF[Domestic_offals] = Population_index_Burkina*(((2*quantity_demanded_BF*(rho_BF^2))-(rho_BF*(Demand_intercept_BF[Domestic_offals]+DGAMMA_BF[Domestic_offals]*Income_index_BF[Domestic_offals])))/DBETA_BF[Domestic_offals])

marginal_revenue_BF[Steaks] = Population_index_Burkina*(((2*quantity_demanded_BF*(phi_BF^2))-(phi_BF*(Demand_intercept_BF[Steaks]+DGAMMA_BF[Steaks]*Income_index_BF[Steaks])))/DBETA_BF[Steaks])

marginal_revenue_GH[Domestic_mixed_cuts] = Population_index_Ghana*((2*quantity_demanded_GH-Demand_intercept_Ghana[Domestic_mixed_cuts]-DGAMMA_GH[Domestic_mixed_cuts]*Income_index_Ghana[Domestic_mixed_cuts])/DBETA_GH[Domestic_mixed_cuts])

marginal_revenue_GH[Domestic_offals] = Population_index_Ghana*(((2*quantity_demanded_GH*(rho_GH^2))-(rho_GH*(Demand_intercept_Ghana[Domestic_offals]+DGAMMA_GH[Domestic_offals]*Income_index_Ghana[Domestic_offals]+DDELTA_Domestic_GH*World_price_offals_in_Cedi)))/DBETA_GH[Domestic_offals])

marginal_revenue_GH[Steaks] = Population_index_Ghana*(((2*quantity_demanded_GH*(phi_GH^2))-(phi_GH*(Demand_intercept_Ghana[Steaks]+DGAMMA_GH[Steaks]*Income_index_Ghana[Steaks])))/DBETA_GH[Steaks])

Maximum_order_processing_rate = Trade_inventory/Order_fulfilliment_time

Monthly_parturition_rate = Annual_parturition_rate/Months

Monthly_parturition_rate_1 = Annual_parturition_rate_1/Months_1

Months = 12

Months_1 = 12

Months_3 = 12

Months_as_female_adult = 132

Months_as_female_adult_1 = 132

Months_as_female_juvenile = 12

Months_as_female_juvenile_1 = 12

Months_as_female_subadult = 36

Months_as_female_subadult_1 = 36

Months_as_male_adult = 72

Months_as_male_adult_1 = 72

Months_as_male_juvenile = 12

Months_as_male_juvenile_1 = 12

Months_as_male_subadult = 36

Months_as_male_subadult_1 = 36

"MR-MC_BF" = sum_marginal_revenue_BF-marginal_cost_BF

"MR-MC_GH" = sum_marginal_revenue_GH-marginal_cost_GH

Net_Burkina_offtakes = Total_marketed_Burkinabe_animals*(1-Percentage_of_offtakes_to_other_West_African_countries)

Net_demand = Excess_supply-Excess_demand

Net_prolificacy_rate = 1

Net_prolificacy_rate_1 = 1

Order_fulfilliment_time = 1

Order_fulfillment_rate = GRAPH(Maximum_order_processing_rate/Excess_demand_from_Ghana)

(0.000, 0.027), (0.100, 0.084), (0.200, 0.141), (0.300, 0.190), (0.400, 0.272), (0.500, 0.391), (0.600, 0.489), (0.700, 0.614), (0.800, 0.734), (0.900, 0.853), (1.000, 1.000)

Other_animals_to_GH = 0

Own_price_elasticity_foreign_offals = -1

Per_capita_income_growth_in_BF = 0.024 {Source: World Bank using ten-year GDP per capita average at constant $}

Per_capita_income_growth_in_Ghana = 0.0439 {Source: World Bank using ten-year GDP per capita average at constant $}

Perceived_trade_inventory = SMTH1(Inventory_coverage, Time_to_perceive_inventory)

Percentage_female = 0.5

Percentage_female_1 = 0.5

Percentage_male = 1-Percentage_female

Percentage_male_1 = 1-Percentage_female_1

Percentage_of_offtakes_to_other_West_African_countries = 0

phi_BF = Reference_demand_in_BF[Steaks]/Reference_demand_in_BF[Domestic_mixed_cuts]

phi_GH = Reference_demand_in_Ghana[Steaks]/Reference_demand_in_Ghana[Domestic_mixed_cuts]

price_equation_BF[Domestic_mixed_cuts] = Population_index_Burkina*Income_index_BF[Domestic_mixed_cuts]*((quantity_demanded_BF-Demand_intercept_BF[Domestic_mixed_cuts]-Population_index_Burkina*DGAMMA_BF[Domestic_mixed_cuts]*Income_index_BF[Domestic_mixed_cuts])/DBETA_BF[Domestic_mixed_cuts])

price_equation_BF[Domestic_offals] = Population_index_Burkina*Income_index_BF[Domestic_offals]*((quantity_demanded_BF*rho_BF-Demand_intercept_BF[Domestic_offals]-Population_index_Burkina*DGAMMA_BF[Domestic_offals]*Income_index_BF[Domestic_offals])/DBETA_BF[Domestic_offals])

price_equation_BF[Steaks] = Population_index_Burkina*Income_index_BF[Steaks]*((quantity_demanded_BF*phi_BF-Demand_intercept_BF[Steaks]-Population_index_Burkina*DGAMMA_BF[Steaks]*Income_index_BF[Steaks])/DBETA_BF[Steaks])

price_equation_GH[Domestic_mixed_cuts] = Population_index_Ghana*Income_index_Ghana[Domestic_mixed_cuts]*((quantity_demanded_GH-Demand_intercept_Ghana[Domestic_mixed_cuts]-Population_index_Ghana*DGAMMA_GH[Domestic_mixed_cuts]*Income_index_Ghana[Domestic_mixed_cuts])/DBETA_GH[Domestic_mixed_cuts])

price_equation_GH[Domestic_offals] = Population_index_Ghana*Income_index_Ghana[Domestic_offals]*((quantity_demanded_GH*rho_GH-Demand_intercept_Ghana[Domestic_offals]-Population_index_Ghana*DGAMMA_GH[Domestic_offals]*Income_index_Ghana[Domestic_offals]-DDELTA_Domestic_GH*World_price_offals_in_Cedi)/DBETA_GH[Domestic_offals])

price_equation_GH[Steaks] = Population_index_Ghana*Income_index_Ghana[Steaks]*((quantity_demanded_GH*phi_GH-Demand_intercept_Ghana[Steaks]-Population_index_Ghana*DGAMMA_GH[Steaks]*Income_index_Ghana[Steaks])/DBETA_GH[Steaks])

price_of_GH_offals_in_CFA = Exchange_rate_GHS:CFA*price_equation_GH[Domestic_offals]

Probability_of_adult_death = Male_adult_hazard_rate_deaths

Probability_of_adult_death_1 = Male_adult_hazard_rate_deaths_1

Probability_of_adult_offtake = Male_adult_hazard_rate_offtakes

Probability_of_adult_offtake_1 = Male_adult_hazard_rate_offtakes_1

Probability_of_female_adult_death = Female_adult_hazard_rate_deaths

Probability_of_female_adult_death_1 = Female_adult_hazard_rate_deaths_1

Probability_of_female_adult_offtake = Female_adult_hazard_rate_offtakes

Probability_of_female_adult_offtake_1 = Female_adult_hazard_rate_offtakes_1

Probability_of_female_juvenile_death = Female_juvenile_hazard_rate_deaths

Probability_of_female_juvenile_death_1 = Female_juvenile_hazard_rate_deaths_1

Probability_of_female_juvenile_offtake = Female_juvenile_hazard_rate_offtakes

Probability_of_female_juvenile_offtake_1 = Female_juvenile_hazard_rate_offtakes_1

Probability_of_female_subadult_death = Female_subadult_hazard_rate_deaths

Probability_of_female_subadult_death_1 = Female_subadult_hazard_rate_deaths_1

Probability_of_female_subadult_offtake = Female_subadult_hazard_rate_offtakes

Probability_of_female_subadult_offtake_1 = Female_subadult_hazard_rate_offtakes_1

Probability_of_juvenile_death = Male_juvenile_hazard_rate_deaths

Probability_of_juvenile_death_1 = Male_juvenile_hazard_rate_deaths_1

Probability_of_juvenile_offtake = Male_juvenile_hazard_rate_offtakes

Probability_of_juvenile_offtake_1 = Male_juvenile_hazard_rate_offtakes_1

Probability_of_subadult_death = Male_subadult_hazard_rate_deaths

Probability_of_subadult_death_1 = Male_subadult_hazard_rate_deaths_1

Probability_of_subadult_offtake = Male_subadult_hazard_rate_offtakes

Probability_of_subadult_offtake_1 = Male_subadult_hazard_rate_offtakes_1

Productivity_shock = IF TIME > 60 THEN Shock_percentage ELSE 1

Quantity_animals_demanded_ROWA = EXP(Demand_intercept_ROWA)*Traded_price^Demand_elasticity_ROWA*(Income_index_ROWA^Income_elasticity_ROWA)*Population_index_ROWA

quantity_demanded_BF = X_BF

quantity_demanded_GH = X_GH

Reference_inventory = 1

Relative_inventory = Perceived_trade_inventory/Reference_inventory

rho_BF = Reference_demand_in_BF[Domestic_offals]/Reference_demand_in_BF[Domestic_mixed_cuts]

rho_GH = Reference_demand_in_Ghana[Domestic_offals]/Reference_demand_in_Ghana[Domestic_mixed_cuts]

Seasonal_offtake_trend = GRAPH(TIME)

(0.0, 1.000), (1.0, 1.090), (2.0, 0.970), (3.0, 1.110), (4.0, 1.000), (5.0, 0.880), (6.0, 0.640), (7.0, 0.840), (8.0, 0.730), (9.0, 1.040), (10.0, 1.240), (11.0, 1.180), (12.0, 1.300), (13.0, 1.090), (14.0, 0.970), (15.0, 1.110), (16.0, 1.000), (17.0, 0.880), (18.0, 0.640), (19.0, 0.840), (20.0, 0.730), (21.0, 1.040), (22.0, 1.240), (23.0, 1.180), (24.0, 1.300), (25.0, 1.090), (26.0, 0.970), (27.0, 1.110), (28.0, 1.000), (29.0, 0.880), (30.0, 0.640), (31.0, 0.840), (32.0, 0.730), (33.0, 1.040), (34.0, 1.240), (35.0, 1.180), (36.0, 1.300), (37.0, 1.090), (38.0, 0.970), (39.0, 1.110), (40.0, 1.000), (41.0, 0.880), (42.0, 0.640), (43.0, 0.840), (44.0, 0.730), (45.0, 1.040), (46.0, 1.240), (47.0, 1.180), (48.0, 1.300), (49.0, 1.090), (50.0, 0.970), (51.0, 1.110), (52.0, 1.000), (53.0, 0.880), (54.0, 0.640), (55.0, 0.840), (56.0, 0.730), (57.0, 1.040), (58.0, 1.240), (59.0, 1.180), (60.0, 1.300), (61.0, 1.090), (62.0, 0.970), (63.0, 1.110), (64.0, 1.000), (65.0, 0.880), (66.0, 0.640), (67.0, 0.840), (68.0, 0.730), (69.0, 1.040), (70.0, 1.240), (71.0, 1.180), (72.0, 1.300), (73.0, 1.090), (74.0, 0.970), (75.0, 1.110), (76.0, 1.000), (77.0, 0.880), (78.0, 0.640), (79.0, 0.840), (80.0, 0.730), (81.0, 1.040), (82.0, 1.240), (83.0, 1.180), (84.0, 1.300), (85.0, 1.090), (86.0, 0.970), (87.0, 1.110), (88.0, 1.000), (89.0, 0.880), (90.0, 0.640), (91.0, 0.840), (92.0, 0.730), (93.0, 1.040), (94.0, 1.240), (95.0,

1.180), (96.0, 1.300), (97.0, 1.090), (98.0, 0.970), (99.0, 1.110), (100.0, 1.000), (101.0, 0.880), (102.0, 0.640), (103.0, 0.840), (104.0, 0.730), (105.0, 1.040), (106.0, 1.240), (107.0, 1.180), (108.0, 1.300), (109.0, 1.090), (110.0, 0.970), (111.0, 1.110), (112.0, 1.000), (113.0, 0.880), (114.0, 0.640), (115.0, 0.840), (116.0, 0.730), (117.0, 1.040), (118.0, 1.240), (119.0, 1.180), (120.0, 1.300)

Seasonal_offtakes = IF Simulation_counter=1 THEN Seasonal_offtake_trend ELSE 1

Share_adult_females = 0.41

Share_adult_females_1 = 0.41

Share_adult_males = 0.08

Share_adult_males_1 = 0.08

Share_juvenile_females = 0.09

Share_juvenile_females_1 = 0.09

Share_juvenile_males = 0.08

Share_juvenile_males_1 = 0.08

Share_subadult_females = 0.2

Share_subadult_females_1 = 0.2

Share_subadult_males = 0.14

Share_subadult_males_1 = 0.14

Shock_percentage = 1

Simulation_counter = 1

smooth_traded_price = SMTH1(Expected_traded_price, 12)

Stochastic_shock_SD = 0.002

sum_marginal_revenue_BF = marginal_revenue_BF[Domestic_mixed_cuts]+marginal_revenue_BF[Domestic_offals]+marginal_revenue_BF[Steaks]

sum_marginal_revenue_GH = marginal_revenue_GH[Domestic_mixed_cuts]+marginal_revenue_GH[Domestic_offals]+marginal_revenue_GH[Steaks]

Supply_elasticity = 1

Supply_elasticity_1 = 1

Survival_rate_adult_females = 1-Probability_of_female_adult_death-Probability_of_female_adult_offtake

Survival_rate_adult_females_1 = 1-Probability_of_female_adult_death_1-Probability_of_female_adult_offtake_1

Survival_rate_adult_male = 1-Probability_of_adult_death-Probability_of_adult_offtake

Survival_rate_adult_male_1 = 1-Probability_of_adult_death_1-Probability_of_adult_offtake_1

Survival_rate_juvenile_females = 1-Probability_of_female_juvenile_death-Probability_of_female_juvenile_offtake

Survival_rate_juvenile_females_1 = 1-Probability_of_female_juvenile_death_1-Probability_of_female_juvenile_offtake_1

Survival_rate_juvenile_male = 1-Probability_of_juvenile_offtake-Probability_of_juvenile_death

Survival_rate_juvenile_male_1 = 1-Probability_of_juvenile_offtake_1-Probability_of_juvenile_death_1

Survival_rate_subadult_females = 1-Probability_of_female_subadult_death-Probability_of_female_subadult_offtake

Survival_rate_subadult_females_1 = 1-Probability_of_female_subadult_death_1-Probability_of_female_subadult_offtake_1

Survival_rate_subadult_male = 1-Probability_of_subadult_offtake-Probability_of_subadult_death

Survival_rate_subadult_male_1 = 1-Probability_of_subadult_offtake_1-Probability_of_subadult_death_1

Time_to_adjust_traded_price = 12

Time_to_perceive_inventory = 1

Total_animals = Total_females+Total_males

Total_animals_1 = Total_females_1+Total_males_1

Total_females = Adult_females+Juvenile_females+Subadult_females

Total_females_1 = Adult_females_1+Juvenile_females_1+Subadult_females_1

Total_males = Adult_males+Juvenile_males+Subadult_males

Total_males_1 = Adult_males_1+Juvenile_males_1+Subadult_males_1

Total_marketed_Burkinabe_animals = Juvenile_sales+Juvenile_female_sales+Adult_female_sales+Subadult_sales+Subadult_female_sales+Adult_sales

Total_marketed_Ghana_animals = Juvenile_sales_1+Subadult_sales_1+Adult_sales_1+Juvenile_female_sales_1+Subadult_female_sales_1+Adult_female_sales_1

Traded_price = Expected_traded_price*Effect_of_inventory_coverage_on_price

Transition_rate_female_adult_to_culled = (Survival_rate_adult_females^(Months_as_female_adult-1)-Survival_rate_adult_females^Months_as_female_adult)/(1-Survival_rate_adult_females^Months_as_female_adult)

Transition_rate_female_adult_to_culled_1 = (Survival_rate_adult_females_1^(Months_as_female_adult_1-1)-Survival_rate_adult_females_1^Months_as_female_adult_1)/(1-Survival_rate_adult_females_1^Months_as_female_adult_1)

Transition_rate_female_juvenile_to_subadult = (Survival_rate_juvenile_females^(Months_as_female_juvenile-1)-Survival_rate_juvenile_females^Months_as_female_juvenile)/(1-Survival_rate_juvenile_females^Months_as_female_juvenile)

Transition_rate_female_juvenile_to_subadult_1 = (Survival_rate_juvenile_females_1^(Months_as_female_juvenile_1-1)-Survival_rate_juvenile_females_1^Months_as_female_juvenile_1)/(1-Survival_rate_juvenile_females_1^Months_as_female_juvenile_1)

Transition_rate_female_subadult_to_adult = (Survival_rate_subadult_females^(Months_as_female_subadult-1)-Survival_rate_subadult_females^Months_as_female_subadult)/(1-Survival_rate_subadult_females^Months_as_female_subadult)

Transition_rate_female_subadult_to_adult_1 = (Survival_rate_subadult_females_1^(Months_as_female_subadult_1-1)-Survival_rate_subadult_females_1^Months_as_female_subadult_1)/(1-Survival_rate_subadult_females_1^Months_as_female_subadult_1)

Transition_rate_male_adult_to_culled = (Survival_rate_adult_male^(Months_as_male_adult-1)-Survival_rate_adult_male^Months_as_male_adult)/(1-Survival_rate_adult_male^Months_as_male_adult)

Transition_rate_male_adult_to_culled_1 = (Survival_rate_adult_male_1^(Months_as_male_adult_1-1)-Survival_rate_adult_male_1^Months_as_male_adult_1)/(1-Survival_rate_adult_male_1^Months_as_male_adult_1)

Transition_rate_male_juvenile_to_subadult = (Survival_rate_juvenile_male^(Months_as_male_juvenile-1)-Survival_rate_juvenile_male^Months_as_male_juvenile)/(1-Survival_rate_juvenile_male^Months_as_male_juvenile)

Transition_rate_male_juvenile_to_subadult_1 = (Survival_rate_juvenile_male_1^(Months_as_male_juvenile_1-1)-Survival_rate_juvenile_male_1^Months_as_male_juvenile_1)/(1-Survival_rate_juvenile_male_1^Months_as_male_juvenile_1)

Transition_rate_male_subadult_to_adult = (Survival_rate_subadult_male^(Months_as_male_subadult-1)-Survival_rate_subadult_male^Months_as_male_subadult)/(1-Survival_rate_subadult_male^Months_as_male_subadult)

Transition_rate_male_subadult_to_adult_1 = (Survival_rate_subadult_male_1^(Months_as_male_subadult_1-1)-Survival_rate_subadult_male_1^Months_as_male_subadult_1)/(1-Survival_rate_subadult_male_1^Months_as_male_subadult_1)

"Transport_margins_BF-GH" = 1.1

USD:CFA_rate = Exchange_rate_USD:GHS*Exchange_rate_GHS:CFA

World_price_offals_in_Cedi = Imported_beef_price_USD*Exchange_rate_USD:GHS

World_price_offals_in_CFA = Exchange_rate_GHS:CFA*World_price_offals_in_Cedi

World_price_offals_to_Northern_Ghana_in_Cedi = World_price_offals_in_Cedi*1.06 }

**Table S1: Disaggregation of beef exports to Ghana by country of origin, 2018**

| Country of origin | Product | Export value (USD) | Export volume (kg) | Unit value (USD/kg) |
|---|---|---|---|---|
| France | Fresh beef | 970 | 128 | 7.58 |
| Italy | Fresh beef | 6,241 | 227 | 27.49 |
| Luxembourg | Fresh beef | 2,036 | 54 | 37.70 |
| South Africa | Fresh beef | 4,584 | 521 | 8.80 |
| United Kingdom | Fresh beef | 32,427 | 25,867 | 1.25 |
| USA | Fresh beef | 3,057 | 356 | 8.59 |
| Botswana | Fresh beef | 73 | 9 | 8.11 |
| Belgium | Frozen beef | 4,202,168 | 3,291,955 | 1.28 |
| Brazil | Frozen beef | 516,768 | 316,789 | 1.63 |
| Canada | Frozen beef | 23,390 | 2,377 | 9.84 |
| France | Frozen beef | 17,203 | 3,294 | 5.22 |
| Germany | Frozen beef | 182,664 | 25,222 | 7.24 |
| Ireland | Frozen beef | 3,379,239 | 2,798,606 | 1.21 |
| Italy | Frozen beef | 655,974 | 847,603 | 0.77 |
| Netherlands | Frozen beef | 1,264,554 | 829,678 | 1.52 |
| Poland | Frozen beef | 523,621 | 572,447 | 0.91 |
| India | Frozen beef | 641,357 | 317,000 | 2.02 |
| South Africa | Frozen beef | 316,429 | 47,322 | 6.69 |
| Spain | Frozen beef | 166,833 | 33,381 | 5.00 |
| United Kingdom | Frozen beef | 1,067,253 | 844,544 | 1.26 |
| USA | Frozen beef | 289,259 | 16,475 | 17.56 |
| Kenya | Frozen beef | 20,627 | 2,300 | 8.97 |
| Ukraine | Frozen beef | 11,759 | 28,000 | 0.42 |
| Argentina | Offals | 327,316 | 318,763 | 1.03 |
| Austria | Offals | 31,495 | 25,600 | 1.23 |
| Belgium | Offals | 4,730,824 | 3,806,161 | 1.24 |
| Brazil | Offals | 5,039,315 | 3,650,443 | 1.38 |
| Croatia | Offals | 77,479 | 125,000 | 0.62 |
| Cyprus | Offals | 22,699 | 24,660 | 0.92 |
| Estonia | Offals | 29,142 | 78,000 | 0.37 |
| France | Offals | 484,377 | 226,367 | 2.14 |

| | | | | |
|---|---|---|---|---|
| Germany | Offals | 2,649,137 | 3,579,817 | 0.74 |
| Greece | Offals | 13,762 | 24,807 | 0.55 |
| Iceland | Offals | 75,490 | 98,780 | 0.76 |
| Ireland | Offals | 8,114,371 | 6,761,650 | 1.20 |
| Italy | Offals | 2,954,252 | 3,229,267 | 0.91 |
| Other Asia, nes | Offals | 3,746 | 5,400 | 0.69 |
| Netherlands | Offals | 6,619,382 | 5,387,134 | 1.23 |
| Norway | Offals | 228,685 | 248,530 | 0.92 |
| Paraguay | Offals | 84,837 | 83,997 | 1.01 |
| Poland | Offals | 742,650 | 904,017 | 0.82 |
| Russian Federation | Offals | 854,487 | 1,053,160 | 0.81 |
| Serbia | Offals | 76,538 | 126,580 | 0.60 |
| South Africa | Offals | 2,233 | 457 | 4.89 |
| Spain | Offals | 1,614,992 | 1,499,750 | 1.08 |
| Sweden | Offals | 86,789 | 101,000 | 0.86 |
| United Kingdom | Offals | 2,795,969 | 2,463,223 | 1.14 |
| USA | Offals | 144,800 | 174,058 | 0.83 |
| Ukraine | Offals | 15,607 | 27,500 | 0.57 |
| **TOTAL IMPORTS** | | **51,148,860** | **44,028,276** | **1.16** |
| **Total low value cuts** | | **11,136,995** | **9,238,700** | **1.21** |
| **Total offals** | | **37,820,374** | **34,024,121** | **1.11** |
| **Percentage of low value cuts and offals in total imports** | | **96%** | **98%** | |

Source: UN Comtrade; shaded figures denote low value cuts. See text for details.

**Table S2: Baseline data used in system dynamics model of Burkina Faso-Ghana trade corridor**

| Data | Value | Source |
|---|---|---|
| Cattle production – Burkina Faso | 10.14 million heads | Extrapolated from *Tableau de bord de l'économie 2016* for 2015 using a 2% annual growth rate |
| Cattle production - Ghana | 1.734 million heads | FAOSTAT |
| Exports to Ghana | 82,700 heads | Uses 2014 figure from *Annuaires 2014* |
| Slaughter weight – Burkinabe domestic production | 240 kg | Ouedraogo (2018) |
| Slaughter weight – traded animals and Ghanaian production | 300 kg | Suleman (2018) and Ouedraogo (2018) |
| Initial domestic consumption of beef – Burkina Faso | • 47.8 million kg low-value cuts<br>• 14.8 million high-value cuts<br>• 11.9 million kg offals | Extrapolated from net domestic availability of animals. Volume of offals estimated at 10.4% of live slaughter weight based on calculation reported in Ouedraogo (2018). Differentiation of high and low value cuts based on AHDB (2020). |
| Initial domestic consumption of beef – Ghana | • 29.5 million kg low-value cuts<br>• 9.5 million high-value cuts<br>• 7.4 million kg offals | As above |
| Offtake rates | 8.9% | Derived from DynMod as a weighted average of all age cohorts |
| Initial traded price of live animals | 1,000 CFA/kg live weight | Based Suleman (2018) and Ouedraogo (2018). Live animal prices in Ghana are increased by 10% to reflect transport costs as noted in Suleman (2018) |
| Initial prices of beef cuts – Burkina Faso | 2,000 CFA/kg (low and high value cuts); 1,000 CFA/kg (offals) | Ouedraogo (2018) |

| | | |
|---|---|---|
| Initial prices of beef cuts – Ghana | 14.3 Cedi/kg (low and high value cuts); 12.1 Cedi/kg (offals) | Suleman (2018) |
| Price elasticities of demand for beef | -2.0 (all cuts) | Assumed for both countries. Monopoly model requires assumption of elastic demand. We remark that short-run elasticities for beef at industry level from Lanfranco and Rava (2014) in Uruguay reveal similar magnitude differences (between -1.4 and -1.9 for cuts, -1.2 for mixed meats) while Taljaard (2003) cites a number of studies whereby own-price elasticities of beef in South Africa are elastic (greater than -1 in absolute value). |
| Income elasticities of demand for beef | +1.0 (low-value cuts and offals); +2.0 (high-value cuts) | Assumed for both countries. |
| GDP per capita growth rate (constant dollars) – Burkina Faso | 2.4% | World Bank, using ten-year average 2008-2017 |
| GDP per capita growth rate (constant dollars) – Ghana | 4.39% | World Bank, using ten-year average 2008-2017 |
| Currency depreciation rate for Ghanaian Cedi | 5% | Assumed for simulation purposes. Average annual depreciation over 2009-2017 of Cedi vs. USD has been 16%, while against Euro and CFA has been 13%. As these figures are noisy, we assume a lower depreciation rate but conduct sensitivity analysis of a higher depreciation rate (10%). |
| Price for imported offals into Ghana | US$1.86/kg | See table 5 for details. |
| USD to GHC exchange rate | 4.6 | Prevailing rate in 2018 |
| GHC to CFA exchange rate | 117 | Prevailing rate in 2018 |
